# The Unease Modulation Model: An Experiential Model of Stress With Implications for Health, Stress Management, and Public Policy

**DOI:** 10.3389/fpsyt.2019.00379

**Published:** 2019-06-07

**Authors:** Joseph Arpaia, Judith P. Andersen

**Affiliations:** ^1^Private Practitioner, Psychiatry and Behavioral Medicine, Joseph P. Arpaia, MD, LLC, Eugene, OR, United States; ^2^Department of Couples and Family Therapy, University of Oregon, Eugene, OR, United States; ^3^Department of Psychology, University of Toronto Mississauga, Mississauga, ON, Canada

**Keywords:** unease modulation, stress, addiction, anxiety, pain, burnout, social systems

## Abstract

Stress has a pervasive, global, and negative influence on individual health. Stress also has negative effects on families, organizations, and communities. Current models of stress are either too general or too detailed to guide effective interventions across the spectrum of medical and social conditions that are stress-related. A new model is needed that explains how stress can have such varied effects and describes how to reduce its harm. The model must also capture both the dynamic nature of stress and its ability to persist and cause chronic effects. The model must guide those who use it in selecting effective interventions and in developing more effective interventions. Ideally, the model will be helpful to people who are experiencing stress and do not have access to professional help. The authors propose a model in an attempt to address the above concerns. The proposed model is called the Unease Modulation Model (UM Model). Briefly, the UM Model separates stress into several elements common to people’s experience. The model describes how these elements interact and how those interactions lead to recurring states that are associated with health or illness. Finally, the model enables the person under stress to identify the elements where they will have the most leverage to evoke change and apply specific, effective techniques for that purpose. While the model is experiential, it is also based on mathematical theories of perception, nonlinear dynamics, neurophysiology, and cognitive psychology. In spite of this underlying sophistication, it can be used by those without a medical education. The proposed model has been taught successfully to patients in a clinical setting. The model is now being used in an international training program with police officers to address the long-term stress associated with the career and reduce decision-making errors regarding use of force. This article introduces the model by defining components based on patient descriptions of stress and integrating those into a formal structure. We then demonstrate how the model can be applied to a number of medical and psychiatric conditions. The article concludes by briefly discussing the model’s application to family and societal stress-related difficulties.

## Introduction. Why Is a New Model for Stress Necessary?

Stress has a significant, negative, and global effect on health. The conditions that are exacerbated by stress include cardiovascular disease, diabetes, anxiety, depression, chronic pain, and addiction (1–7). Stress also has negative effects on family relationships and developmental processes (8, 9). Stress impairs the function of organizations due to staff burnout, and it increases social unrest in communities and societies (10, 11).

Stress is a nonspecific term used interchangeably to describe both external demands (e.g., giving a speech) and internal processes (e.g., feelings of tension while giving the speech). These internal processes cause physical changes that accumulate over time and have been defined by researchers as “allostatic load,” or the wear and tear on the body (12). Colloquially, stress is treated with stress reduction, with methods including mindfulness, yoga, biofeedback, hypnosis, music, exercise, writing, and pass/fail grading (13–17). The benefits of these methods are limited and difficult to correlate with proposed mechanisms (18, 19). The lack of precision regarding the nature of stress may be one cause of the limited benefits.

**Figure 1:**
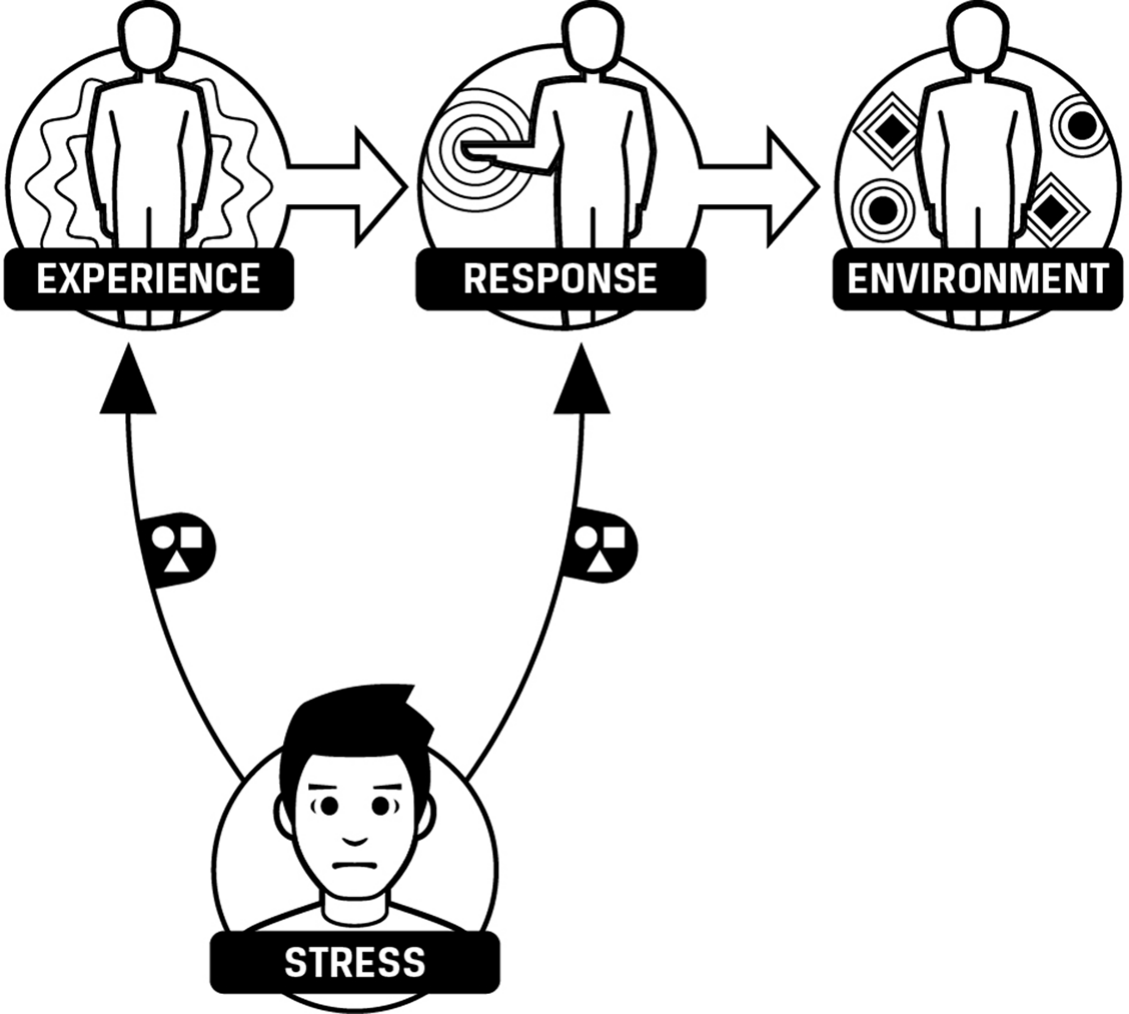
Environment, experience, and response influenced by stress. Stress has an effect on experience and response. The effect is variable as stress can cause benefit or harm.

Existing scientific models of stress, such as the neurovisceral integration model (20–22) and the polyvagal theory (23–25), focus on biochemical mechanisms that are important to researchers but that are far removed from the experience of the person suffering from stress (26, 27). These models use one word “stress” to predict and influence patterns of experience and response by using stress as a control variable (**Figure 1**). Unfortunately, stress, as explained by existing models, has an inconsistent relationship with experiences and responses, sometimes negative and sometimes positive. Subsequently, stress is not useful as a control variable as people experience it in daily life. A model that is both precise enough to direct interventions effectively and simple enough to be used without specialized training is called for. Such a model may be particularly useful in settings where few skilled practitioners are available (28).

The first author has been working with patients for over 20 years in an outpatient psychiatry/addiction medicine practice, treating patients from a wide socioeconomic spectrum who were suffering from a range of psychiatric and comorbid medical conditions. In order to help patients benefit more quickly, he developed and refined a model that builds on scientific principles revealed by prior research [see Refs. (22, 25)]. The model proposed in this paper separates what is commonly called “stress” into component parts that people can understand and treat without needing a laboratory setting or sophisticated equipment.

## Defining Model Components

The model breaks stress into five components, two of which are experienced as external influences and three of which are experienced as internal effects.

- Internal effects
  - Sympathetic nervous system activation (SMP)
  - Parasympathetic nervous system activation (PMP)
  - Reserves
- External influences
  - Difficulty
  - Unease

### Internal Effects: Sympathetic Nervous System, Parasympathetic Nervous System, and Reserves

“I am pushing myself hard.”

Patients use statements like this to describe how they have to exert effort or “push” in order to respond to their environment. Their description of having to push includes symptoms such as increased muscle tension, shallower or more rapid breathing, sweating, rapid heartbeat, tremors, or gastrointestinal distress. These symptoms are consistent with increased sympathetic activation. This component of stress is designated as “SMP.”

“I feel drained.” “I am exhausted.”

As patients continue to push themselves over days or weeks, they often describe feeling like some reserves of energy are depleted or drained. This suggests that “reserves” is another component of stress and “reserves” has an inverse relationship with stress. Lower reserves are associated with higher perceived stress.

“I need a break.” “I need to recharge.”

As patients feel drained, they report a need to rest or recharge their reserves. The physical symptoms associated with resting or recharging include slower and deeper breathing, relaxed muscles, warm hands, and lower heart rate. These are associated with parasympathetic activity, and therefore a third component of stress will be denoted “PMP.” PMP recharges reserves that have been drained by SMP.

**Figure 2:**
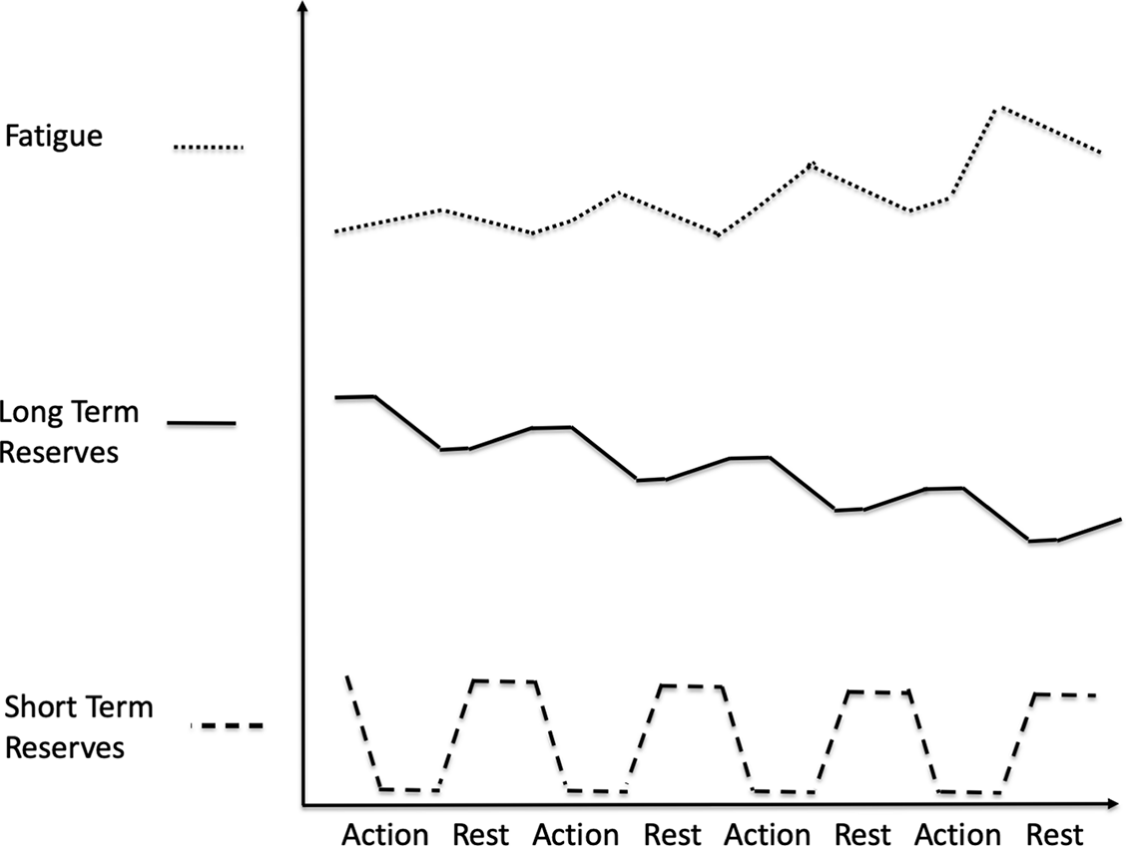
Gradual depletion of long-term reserves. When short-term reserves are depleted, we draw on long-term reserves. Fatigue increases as short-term reserves drop and continues to increase as we draw on long-term reserves. When we rest, we recharge short-term reserves and fatigue drops, but we may not fully recharge long-term reserves. If this happens consistently, then over time long-term reserves become depleted. As long-term reserves get depleted, fatigue increases more sharply when we run out of short-term reserves.

People often experience decreases in energy, or increases in fatigue, in two phases. At first, the person experiences fatigue increasing slowly, then suddenly they experience a sharp increase in fatigue and may even feel suddenly exhausted. This can be modeled by two types of reserves, short-term reserves and long-term reserves (**Figure 2**).

Fatigue increases slowly as short-term reserves drop. But as those become depleted, energy must come from long-term reserves. If long-term reserves are full, then they can continue to supply the needed energy and fatigue continues to increase slowly, but if long-term reserves are low, then there is little energy available in them, and when short-term reserves are exhausted, the increase in fatigue is large.

When people rest, or increase PMP, they replenish both short-term and long-term reserves. However, short-term reserves are replenished at a faster rate than long-term reserves. The replenishment of short-term reserves (e.g., taking a nap or restful sleep) enables the person to return to activity quickly. However, without sufficient rest, long-term reserves are still being drained. If person continues to replenish short-term reserves without fully replenishing long-term reserves for an extended period of time (e.g., insomnia), then the person’s long-term reserves become depleted (**Figure 2**). Very long periods of rest, sometimes months, may be needed to replenish the person’s depleted long-term reserves. This is seen in people who suffer from some medical conditions, e.g., chronic fatigue syndrome, cancer-related fatigue, some forms of depression, and burnout.

**Figure 3:**
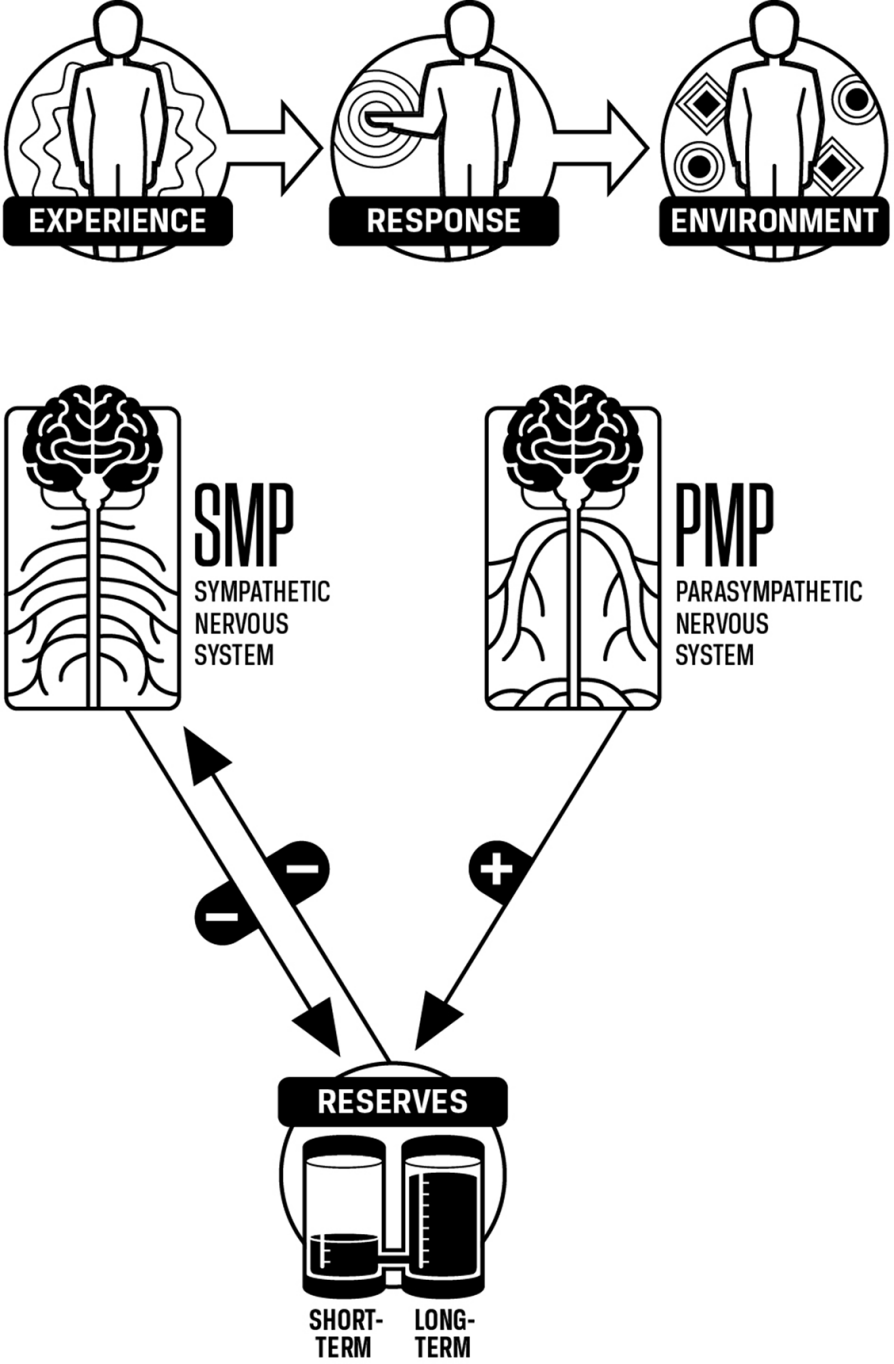
SMP, PMP, and reserves. Increases in SMP decrease reserves and increases in PMP increase reserves. Increases in reserves allow SMP to decrease as less SMP is needed to mobilize energy when reserves are high.

While some patients are more aware than others of the sensations associated with SMP, reserves, and PMP, all can identify the components enough to distinguish them. While these terms refer to consistent subjective experiences, the specific physiologic processes associated with these terms will necessarily change with the situation. For example, the physiologic processes associated with SMP, reserves, and PMP involved in running a race are different from those involved doing customer service and different again from those involved in fighting off an infection. The subjective sense in all these is similar, i.e., pushing (SMP), feeling drained (reserves), or recharging (PMP), and using the same terms helps the patient learn to identify those components in various situations and modulate them more effectively (**Figure 3**).

#### Interaction Between Reserves and SMP

**Figure 4:**
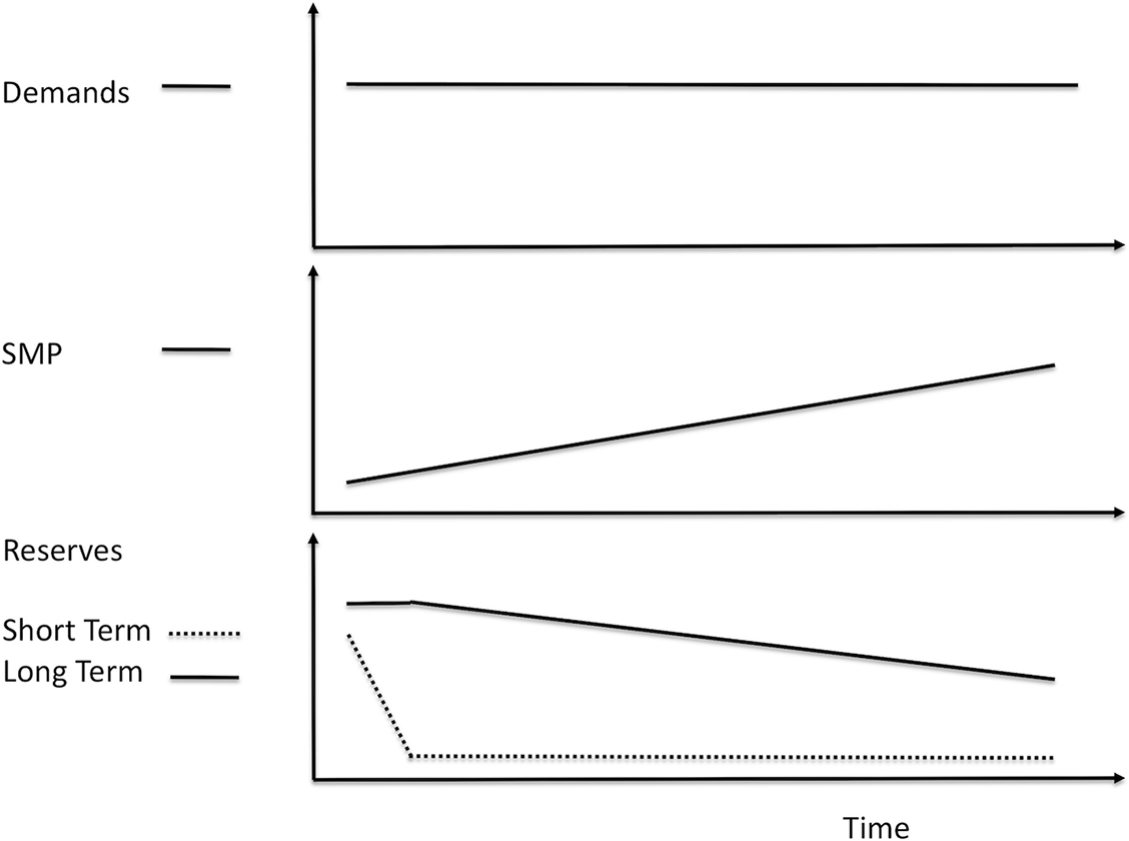
Decreasing reserves increases sympathetic nervous system activation (SMP). As we push to meet demands, short-term reserves decrease. As these decrease, SMP increases. As we continue to meet demands without resting, short-term reserves become depleted. We then use long-term reserves, and as these decrease, SMP continues to increase. These increases in SMP occur even if the demands are not increasing.

The level of SMP is affected by the level of reserves. For example, if a person has to work through the day without having a chance to rest, the person will experience having to push themselves harder throughout the day simply to maintain the same pace. Conceptually, their energy reserves are decreasing, and as their reserves decrease, the person feels more drained. A key point is that as energy reserves drop, SMP must increase even if the demands do not increase (**Figure 4**).

If a person is consistently unable to rest enough to replenish their reserves, then SMP will tend to be chronically high. Conditions characterized by deficits in rest, such as insomnia, are associated with poor health as well as physiologic changes suggesting excessive sympathetic tone, i.e., high SMP (12). The inverse relationship between the level of reserves and the level of SMP may be an explanation for this finding and highlights the importance of implementing methods to maintain adequate reserves. If the person rests briefly at frequent intervals, each period of rest will replenish reserves, keeping SMP from having to increase as much.

### External Influences: Difficulty

“I am under a lot of stress.”

We routinely assess the demands we face with the resources available to meet those demands. The comparison of demands vs. resources can be called difficulty.

- Difficulty is low when demands are lower than resources.
- Difficulty is high when demands are higher than resources.

Note that the assessment process may not be accurate. Often, difficulty is assessed as high because a resource is overlooked, or a demand is overestimated. Perfectionists tend to overestimate the demands they face. Instead of doing “good enough,” they think they have to do flawlessly and assess difficulty as far greater than is accurate.

When demands and resources are approximately equal, then small changes in either can cause large changes in difficulty. For example, if at max a person can carry 50 kg, then increasing the load from 48 kg to 51 kg will cause an enormous increase in difficulty. Colloquially, this is referred to as “the straw that broke the camel’s back,” a large increase in difficulty from a small change in demands.

Demands and resources can also be grouped into various types:

- physical
- cognitive
- social
- financial
- temporal

Certain types of resources, such as social or financial, can be applied to a wider variety of demands. For example, if one’s physical resource for lifting a weight is a maximum of 50 kg, then physical resources are insufficient to lift an object weighing 80 kg. However, cognitive resources might enable one to use a lever to lift the object. Social resources would enable one to get other people to help, and financial resources could be used to pay someone to lift the object.

**Figure 5:**
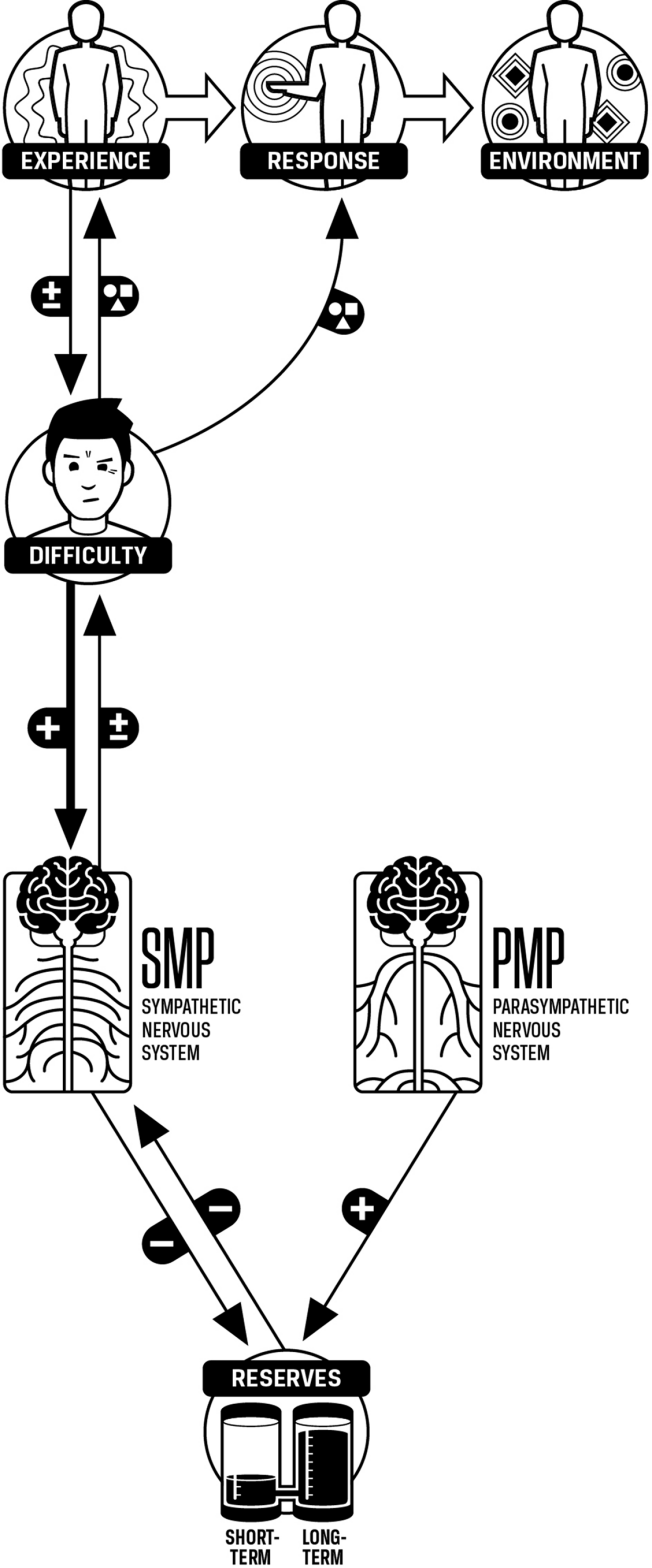
Difficulty, SMP, PMP, and reserves. Experience and response are qualitative phenomena and do not simply increase or decrease. Difficulty changes experience and response in a qualitative (nonnumerical) manner. This is represented by the circle, square, and triangle graphic on the arrows from difficulty to experience and response. Increases in difficulty increase SMP. Experience can increase or decrease difficulty. Increases in SMP can increase or decrease difficulty.

**Figure 6:**
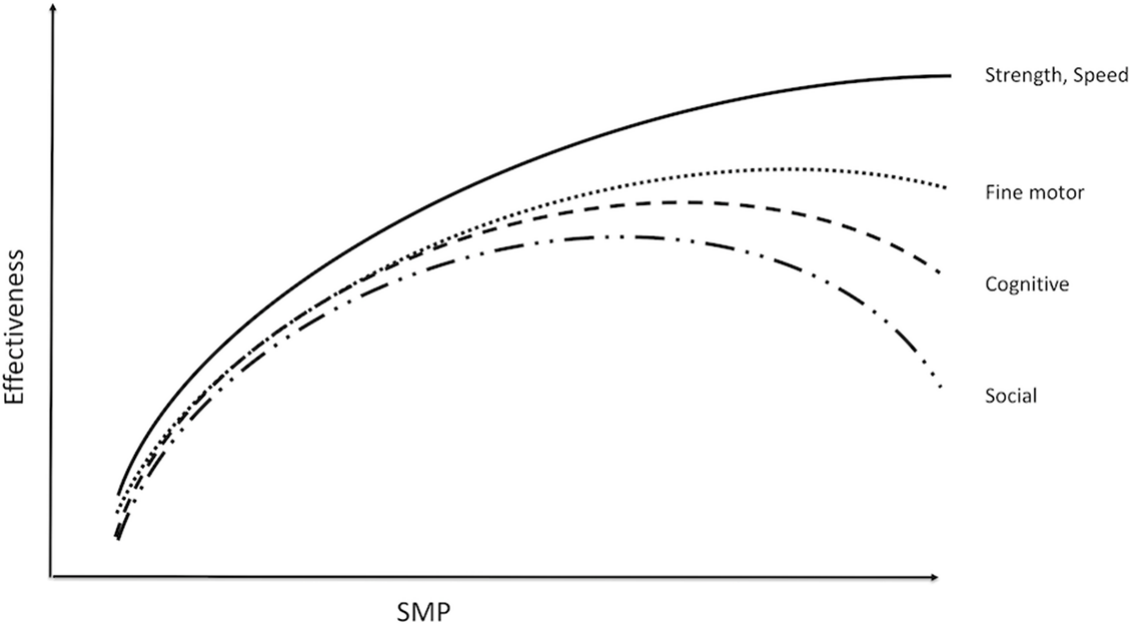
SMP and effectiveness of resources. As SMP increases, effectiveness increases initially for all types of resources. However, the effectiveness of resources other than raw strength and speed decreases as SMP continues to increase. Increases in PMP will shift the peak of the curve to the right, thus maintaining maximal effectiveness at higher levels of SMP.

SMP has a complex effect on difficulty. As SMP begins to increase, resources become more effective, causing difficulty to decrease. However, as SMP continues to increase, certain resources become less effective. This will cause the difficulty associated with those resources to increase (**Figure 5**). For example, raw physical resources such as strength and speed are not significantly reduced, if at all, as SMP increases. However, fine motor skills become reduced at high levels of SMP. Cognitive skills are more sensitive to increases in SMP, and social skills are most sensitive (**Figure 6**).

Increasing PMP reduces the negative effect of SMP and improves effectiveness. Patients who learn techniques to increase PMP have reported an increase in effectiveness during difficult situations.

When a person has to meet several types of demands at once, the SMP required to optimize resources for one type of demand may be excessive for another type of demand. For example, first-responder teams are faced with both physical and cognitive demands. The high level of SMP required for the physical demands can interfere with cognitive and social resources, thus impairing their ability to think and communicate effectively. Increasing PMP in these situations will preserve cognitive and social resources.

### External Influences: Unease

“I feel stressed”

Unease is a component of stress that often accompanies difficulty but also acts independently. The following thought experiment can be used to illustrate the difference between difficulty and unease. Imagine a long plank of wood, 30 m long, 0.5 m wide, and 10 cm thick. The task is to walk the 30 m length of the plank.

Condition 1—The plank is lying on the ground.

Condition 2—The plank is firmly supported at each end but is 100 m above the ground.

The plank is the same width, and therefore the difficulty of the task as defined by demands vs. resources is the same. The increase in elevation in condition 2 creates unease.

This thought experiment also demonstrates a relationship between attention, unease, and SMP. In condition 2, attention is focused on thoughts or images of falling, increasing unease. The increased unease is accompanied by marked increases in SMP. The increases in SMP may be so strong that one’s ability to balance will be compromised. The high unease has caused autonomic changes that then have increased difficulty. If the person focuses their attention on the plank in such a situation, unease will decrease. SMP will not increase as much, and the ability to balance will not be compromised and difficulty will remain low.

Unease occurs when we are aware of a memory, present experience, or expectation in which something we desire is absent or something aversive is present. Unease is dependent on values, preferences, and dislikes and varies significantly between individuals. Thus, what causes unease in one person may reduce it in another. For example, saving money for retirement instead of spending it on a vacation can reduce unease in one person, but can increase unease in another. This can be a problem if the two people are married to each other. People also change the values they prioritize depending on context. Changes in context can modify the level of unease. Doing a task for someone we like is associated with a lower level of unease than doing the same task for someone we dislike. **Table 1** lists experiences associated with increased or decreased unease.

**Table 1:** Types of experiences that increase or decrease unease.

Increase unease	Decrease unease
Discomfort	Comfort
Anticipated aversive outcome	Anticipated desired outcome
Remembered aversive outcome	Remembered desired outcome
Loss of desired object	Gain of desired object
Uncertainty	Belief (even if belief is incorrect)
Rejection	Acceptance by others
Isolation	Connection with others
Being “wrong”	Being “right”
Violating one’s values	Adhering to one’s values (even if doing so causes pain)

The literature on stress often does not distinguish between difficulty and unease. Few research studies in the field of psychophysiology determine how much of the subject’s physiologic response is due to unease and how much is due to the difficulty of the situation studied. In the clinical setting, patients entering treatment also confuse the two. This is problematic because what reduces unease may not reduce difficulty. Patients consistently report that learning to distinguish unease from difficulty and focusing their attention on reducing difficulty rather than unease enables them to make more effective decisions.

#### Unease, Sympathetic Nervous System Activation, and Parasympathetic Nervous System Activation

Patients describe it is hard to relax when uneasy. Conversely, if they are relaxed, they tend to feel less unease. This subjective experience suggests that unease and PMP inhibit each other. As unease increases, PMP decreases, and as PMP increases, then unease decreases.

Patients also describe increases in SMP when they feel uneasy, suggesting that SMP increases as unease increases. The effect of SMP on unease is variable and depends on where attention is focused. If SMP increases and attention is focused on what reduces unease, such as resources, desired outcomes, or the memory of successful responses, then unease will decrease. If attention is focused on what causes unease, then unease can increase further, causing an escalating cycle that is present in many anxiety disorders.

#### Unease vs. Emotion

Unease is associated with some emotions, but unease itself is not an emotion. When unease is present, the emotion experienced is related to the context in which the unease arises.

- Unease about loss is associated with sadness.
- Unease about safety is associated with fear.
- Unease about injustice is associated with anger.
- Unease about not being able to complete a task is associated with frustration.
- Unease about damaging a relationship is associated with guilt.

The specific emotion can be thought of as a perceptual inference influenced by the amount of unease and the context in which the unease is present. The level of unease contributes to the intensity of the emotional experience, with higher levels of unease increasing the intensity. Note that unease can be associated with positive emotions. Excitement occurs when unease is present with the expectation of a desired outcome.

#### Unease and Pleasure

Pleasure comes from obtaining what we desire or avoiding what we are averse to, i.e., pleasure comes from a reduction in unease. In the language of operant conditioning, a decrease in unease is a positive reinforcer and an increase in unease is a negative reinforcer. The larger and faster the change in unease, the stronger the reinforcement (29).

- A response that reduces unease will be positively reinforced and become more likely.
- A response that increases unease will be less likely.

Note that a response that reduces unease will be positively reinforced and become more likely, even if it increases difficulty. For example, in a college student population, learning strategies seemed to be chosen more because of their effect on unease than their effect on learning the material (30).

#### Unease and Habits

A response that reduces unease quickly becomes more likely, and as the response is repeated, that likelihood increases until the response becomes automatic. We can call such automatic responses “habits.” Some habits, like filing important papers instead of leaving them scattered, decrease difficulty and can be called helpful habits. Other habits, like procrastination, increase difficulty and can be called unhelpful habits (**Table 2**).

**Table 2:** Defining behaviors based on changes in difficulty and unease.

	Difficulty decreases	Difficulty increases
Unease decreases	Helpful habit	Unhelpful habit
Unease increases	Helpful behavior	Unhelpful behavior

**Figure 7:**
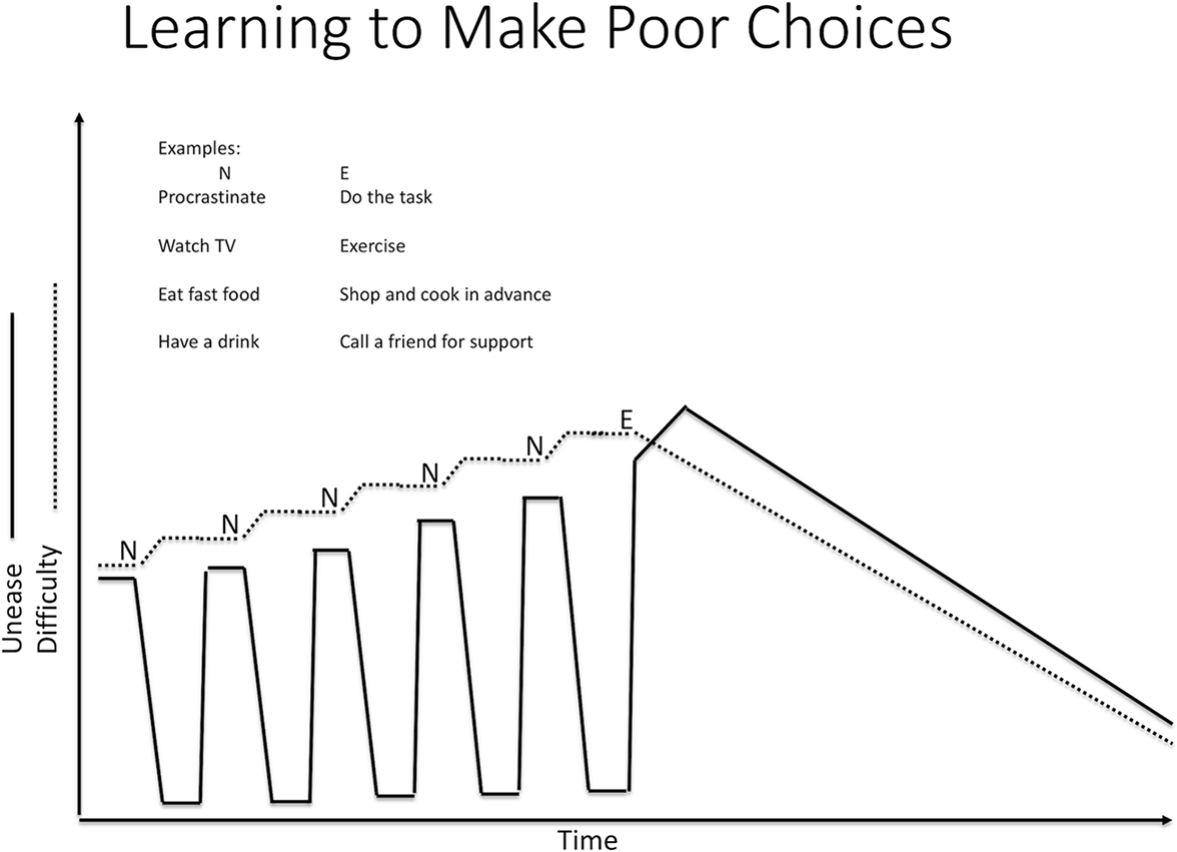
Unease and habits. N is an ineffective response. It reduces unease (desire or aversion) but not difficulty. When we do not deal with difficulty effectively, difficulty tends to increase. E is an effective response. It reduces our difficulty, but at firast, it increases unease since we have to face what we are averse to, or deny ourselves what we desire. But because N reduces unease quickly, our brain learns to choose even though it makes our situation worse. N is learned. E is not.

A “helpful behavior” can be defined as a response that decreases difficulty but that does not reduce unease quickly; in fact, unease may be temporarily increased. For example, someone who is not used to exercising will tend to feel an increase in unease when starting to exercise. It takes effort to select such responses. Because such behaviors do not reduce unease quickly, they tend not to be positively reinforced, i.e., they do not become habits. That is why we can select a helpful behavior several times, but still keep slipping back into selecting an unhelpful habitual response. The fact that behaviors that reduce unease will tend to be selected even if they increase difficulty is one of the ways that stress characterized by chronic unease has a negative impact on health (**Figure 7**). Chronically high unease increases the drive to engage in behaviors that reduce unease quickly, even if they increase difficulty. This is especially relevant for those suffering from behavioral and substance use disorders, as will be discussed below.

##### Discipline vs. Punishment

Discipline is a process of engaging in a helpful behavior and tolerating the initial increase in unease for the purpose of reducing difficulty. The reduction in difficulty eventually reduces unease, but that may take days. The general principle underlying discipline involves teaching someone to tolerate an initial increase in unease and experience the eventual reduction in unease as pleasurable.

- Discipline involves teaching “unease tolerance.” With repeated practice of the helpful behavior, the brain learns to anticipate the eventual reduction in unease, and the helpful behavior becomes a helpful habit.

Punishment is fundamentally different from discipline in that punishment involves setting up constraints, so the person experiences more unease unless they avoid the behavior that needs to be changed (e.g., illicit drug use). The person will then avoid that behavior but is not learning to tolerate unease. The person is still selecting behaviors to reduce unease quickly. When the external constraint is removed, the person is likely to revert to the undesired behavior, as can be observed in those dealing with addiction or in criminal recidivism.

##### Contentment

When unease decreases, we experience pleasure, and if the unease becomes unnoticeable, then we feel content. However, since unease is unnoticeable, then we cannot experience a reduction in unease. This means that while becoming content is pleasurable, contentment does not remain pleasurable, i.e., contentment is unstable.

- Prolonged contentment leads to boredom because we cannot reduce unease when we are content. The fact that contentment is unstable helps keep us from being satisfied with the status quo. Instead, we become uneasy with our content state and engage in behaviors to reduce that unease.

If we have developed healthy habits, we will tend to engage in activities that are helpful. We will explore, discover, or invent. However, our unease at being content does not always motivate us to engage in activities that reduce difficulty. Prolonged contentment, i.e., boredom, can be a risk factor for unhealthy behaviors.

#### Overview of the Unease Modulation Model

**Figure 8:**
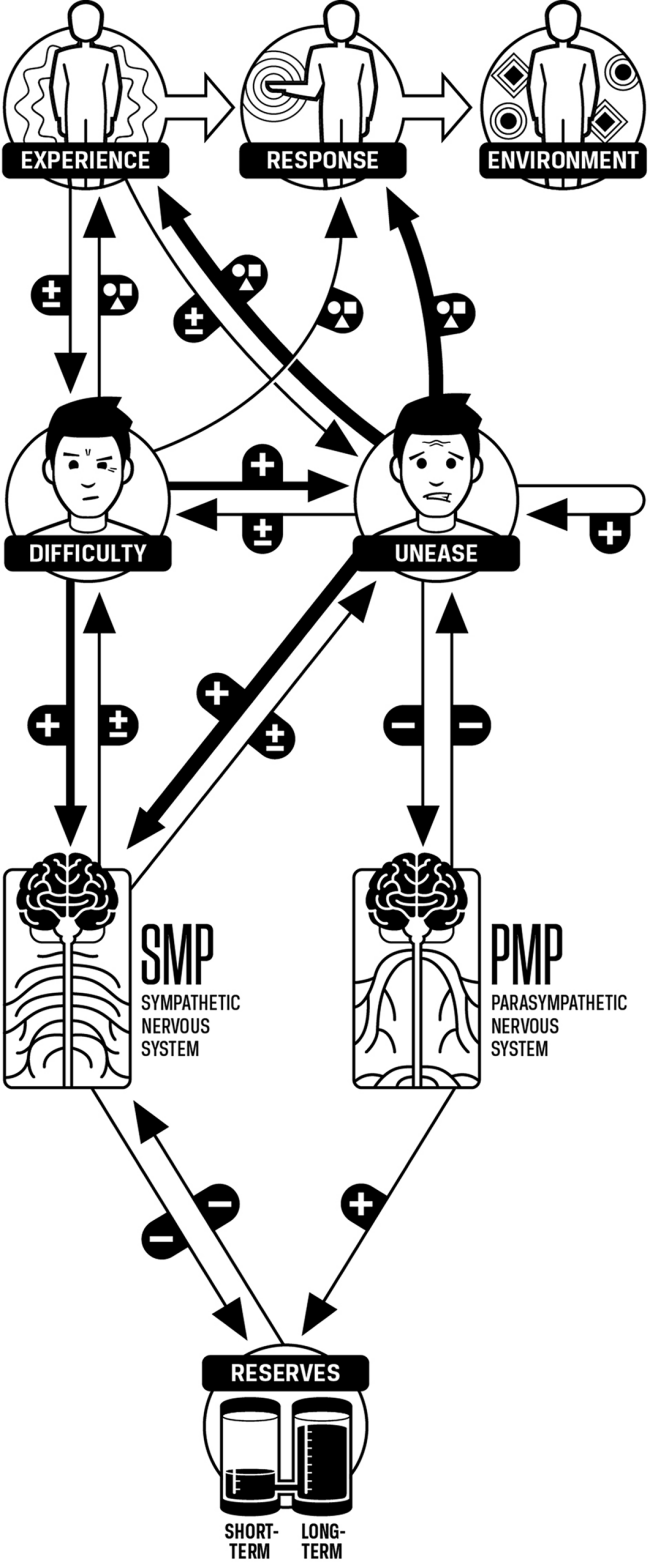
Unease modulation model. Experience and response are qualitative phenomena and do not simply increase or decrease. This is represented by the circle, square, and triangle graphic. Unease has more influence on experience and response than does difficulty. Unease, difficulty, SMP, PMP, and reserves are quantitative, and increases or decreases in these components are indicated by the “+” and “-” graphics. Increases in unease can increase or decrease difficulty. Increases in unease cause larger increases in SMP than difficulty. Increases in unease cause decreases in PMP, and increases in unease can cause further increases in unease. Experience has an increasing or decreasing effect on unease. Increases in SMP will also increase or decrease unease. Increases in difficulty tend to increase unease. Increases in PMP will decrease unease.

The model now has the necessary components and interactions to explain stress and its effects. Note that unease acts as a hub of the model. It affects other components strongly and is affected strongly by those components. In particular, the interactions among unease, SMP, PMP, and reserves can create reinforcing feedback loops that can drive the system to pathological states. Unease also drives response selection and can thus cause the automatic selection of responses that increase difficulty and are unhealthy (**Figure 8**).

A proposed mathematical expression of the UM Model is given in the supplement. Briefly, the components of stress and their associated processes are an example of a nonlinear dynamical system. The relationships among the components give rise to the attractor states of the system. These attractor states manifest as consistent patterns of perception, internal processing, and responding. Personality traits and disorders are examples of attractor states. Variations in the sources of unease, difficulty, and response selection give rise to different personality traits and disorders. For example, the Cluster C Personality Disorders are:

- *Avoidant Personality Disorder:* “A pervasive pattern of social inhibition, feelings of inadequacy, and hypersensitivity to negative evaluation” (31, p. 672). The UM Model explains this as high levels of unease at the possibility of being rejected, leading to avoidant behaviors to reduce that unease. These avoidant behaviors make social interactions more difficult and increase the possibility of being rejected. This makes unease even higher and intensifies the avoidant behaviors.
- *Dependent Personality Disorder:* “A pervasive and excessive need to be taken care of that leads to submissive and clinging behavior and fears of separation” (31, p. 675). In this case, the person assesses the difficulty of taking care of oneself as high and is uneasy about that. The submissive behavior reduces the unease because the submissive behavior allows other people to act as resources, thus reducing difficulty. The person does not develop their own resources, so assessed difficulty remains high and unease continues to be high as well, reinforcing the clinging behavior.
- *Obsessive-Compulsive Personality Disorder:* “A pervasive pattern of preoccupation with orderliness, perfectionism, and mental and interpersonal control, at the expense of flexibility, openness, and efficiency” (31, p. 678). In this case, unease about uncertainty is high and the person has learned that responses to increase order and control reduce that unease. These responses become generalized and are used to deal with unease from all sources. Efforts to increase control often make situations more difficult, and the person becomes uneasy. Since the response to unease is to exert control, the person becomes more controlling, making the situation even more difficult.

The appraisal of unease varies on the time scale of shifts in attention and thought. This means the system is highly dynamic, in which components and responses can change in a fraction of a second. The dynamic nature of the system enables it to function effectively in an environment that can change rapidly. It also means that the timing of interventions can affect their effectiveness. Since attention has a significant effect on unease, methods for training attention can increase the ability to modulate unease, thus reducing its negative effects.

Interventions based on the UM Model that are used to reduce stress will tend to follow some simple principles but will need to be adapted to meet the specific manifestations of each individual’s condition.

## Non-Clinical Applications

The UM Model, as described above, was developed and refined in a clinical setting. The model has been used to individualize treatment interventions for patients over many years. More recently, the model was tested in a nonclinical setting with law enforcement officers. Applications of the model, to reduce stress and improve health among nonclinical populations, are a test of how the model can be applied by the layperson in nonclinical settings.

Police officers routinely encounter situations that are defined by high difficulty and high unease (e.g., a high-speed car chase and a violent domestic abuser). The model predicts that the police officer will experience high SMP. Research indicates that this is indeed the case, particularly when officers face potentially confrontational encounters where the application of force may be necessary (32–34). The police profession is one in which there are real environmental threats that the officer must assess and attend to, simultaneously considering the legal and ethical ramifications of their actions. Thus, police officers must maintain PMP in the presence of SMP activation in order to have the cognitive and social skills necessary to de-escalate threat and resolve confrontational issues.

Police training has traditionally focused on weapons and the use of force. This focus on weapons and force overlooks the fact that during encounters with the public, high levels of SMP activation will inhibit the cognitive and social skills related to de-escalation (35, 36). To address the gap in police training, the authors applied the model to an intervention for police that focused on reducing lethal force errors by modulating SMP and PMP activation (33). Using heart-rate variability biofeedback, the researchers taught officers how to activate PMP in the presence of SMP and how to integrate this into their scenario-based training, thus programming the techniques to become automatic responses (33). The longitudinal study followed officers for 18 months after the initial intervention training. Dramatic reductions in lethal force errors were observed across every retention test (6, 12, and 18 months).

The take home message is that a simple reduction in SMP will not improve performance among police officers. In potentially threatening encounters, SMP is necessary. However, increasing PMP modulates the impact of SMP on performance. Specifically, the PMP blocks the suppression of cognitive and social skills (e.g., de-escalation) during high SMP activation. So, an individual is still able to think clearly and apply their training and logic in times of high SMP activation.

High PMP without SMP would be potentially dangerous in policing in that officers may miss threat cues that cost the lives of self or others or bring harm (37). Thus, it is a misconception that officers need to be relaxed during an encounter in order to improve de-escalation skills. While it is true that as anyone in a stressful job does need to relax in order to refill reserves, occupationally relevant tasks in policing require both SMP and PMP activation.

## Therapeutic Interventions

**Figure 9:**
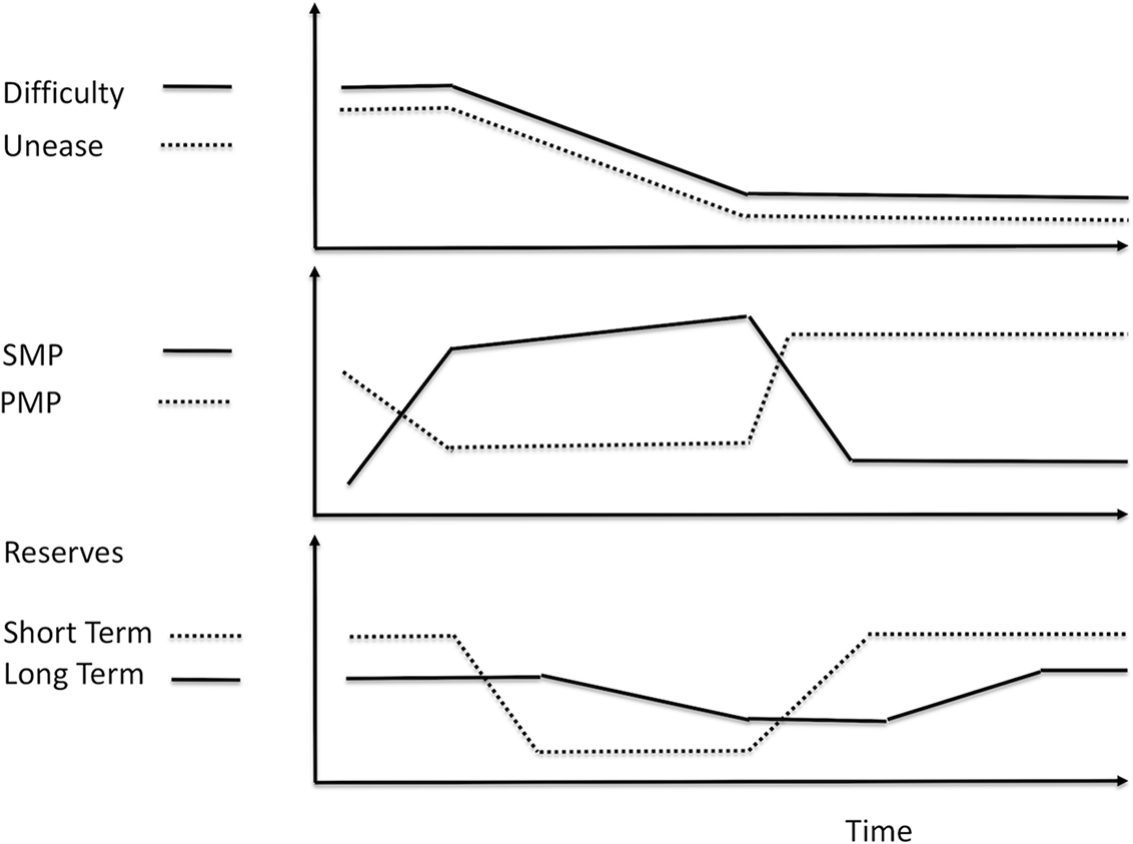
Normal response to difficulty. When difficulty is high because of demands, then unease tends to be high as well. SMP increases and unease reduces PMP. Energy is drawn from short-term and then long-term reserves to mobilize resources to meet the demands. As the demands are completed, difficulty decreases and unease also decreases. This allows SMP to decrease. PMP increases and replenishes reserves. The system is ready for the next difficulty.

What people describe as “good stress” occurs when the following conditions are met (**Figure 9**):

- difficulty is assessed accurately so resources are allocated to meet demands effectively,
- unease is appraised so that it is positively correlated with difficulty,
- the unease helps attention focus on resources and helpful responses,
- increases in unease cause large enough increases in SMP so that the effectiveness of resources increases,
- PMP is maintained so that large increases in SMP do not reduce effectiveness of resources,
- as demands are met, difficulty and unease decrease,
- the decrease in unease reduces SMP and increases PMP,
- the increase in PMP replenishes reserves back to their original levels.

When one or more of these conditions are not met, the different components can interact to create various feedback loops that cause symptoms. In particular, the interactions among unease, SMP, and PMP are mutually reinforcing, and thus, changes in one of these components can cause rapid destructive changes in the other two, with subsequent negative effects on the rest of the system. Several methods, as reviewed below, can be used to reduce the problematic feedback loops that cause symptoms.

### Therapeutic Relationship

A therapeutic relationship between the patient and the clinician will reduce unease in the patient due to the effect of social connections on unease. The reduction in unease from the therapeutic relationship can enable the patient to face situations that would otherwise have caused intolerable levels of unease. By facing such situations, the patient learns to handle them more effectively. Over time, the patient transitions from the relationship with the clinician.

- If the reduction in unease that comes from the therapeutic relationship is simply used as a comfort rather than as an aid to learning, then the patient risks becoming dependent on the clinician and this will tend to interfere with helpful changes.

If the patient is not making progress toward therapeutic goals, then the clinician will need to increase the patient’s unease during sessions in the service of helping the patient learn.

### Relaxation and Mindfulness

Patients who practice relaxation, mindfulness, and similar techniques report a reduction in unease during practice. Physiologic measures suggest that this is accompanied by a reduction in SMP and an increase in PMP (38). Such changes in SMP and PMP are associated with improved health. According to the UM Model, such autonomic changes will also increase reserves and increased reserves will tend to reduce levels of SMP.

- However, relaxation techniques have limited beneficial effect on environmental factors, or on assessment, appraisal, and response-selection processes. If any of these are causing excessive unease, then those will increase SMP, reduce PMP, and nullify the effect of the relaxation techniques.

One recent version of mindfulness is a method called bare attention, which involves acceptance of one’s experience observing phenomena without judgment (39). This can be thought of as an inhibition of assessment and appraisal processes. Since assessment and appraisal are often overused, giving rise to difficulty and unease in situations where neither is helpful, inhibiting assessment and appraisal will tend to reduce unease and thus reduce SMP and increase PMP. Acceptance, or bare attention, may give the same benefits as relaxation methods (40). In clinical practice, bare attention is helpful, but people also need to judge their experience, i.e., use assessment and appraisal, in order to respond effectively to their environment (41).

### Metacognitive Skills

According to the Oxford English Dictionary, metacognition is defined as the awareness and understanding of one’s own thought processes. From the perspective of the UM Model, metacognitive skills enable one to be aware of and understand the components and processes in the model in such a manner as to influence them. The metacognitive skills associated with the UM Model are related to skills such as emotional regulation and cognitive reappraisal. Specific skills associated with the model include

- Manage the assessment of experience to correctly identify demands and resources (42).
- Regulate the appraisal of experience so the intensity of unease is manageable. This can be done using attentional techniques or cognitive techniques (43, 44).

The UM Model distinguishes difficulty from unease. If the level of appraised unease does not match the level of assessed difficulty, then a person may ignore one or the other. Two additional metacognitive skills can help prevent this from happening:

- Assessing the appraisal process,
- Appraising the assessment process.

The first skill requires the person to direct attention to the values and goals that were involved in the appraisal, identify how those were prioritized, and make sure all values and goals that are important to them are involved in the appraisal process. The person “thinks about their feelings.” The second skill is more challenging and requires the person to “feel about their thinking.” The person directs attention at the way they assessed demands and resources sensing for any unease about how the assessment process was carried out. Unease will tend to increase if the assessment process was not thorough or accurate. Typically, the person will ignore that unease, but the metacognitive skills enable them to use the unease to form a more accurate assessment.

The application of metacognitive techniques requires effort and skill, and patients often need guidance to apply them effectively. Evidence exists that metacognitive skills can be trained (45, 46).

Excessive SMP and low PMP can interfere with the cognitive processes required for applying reassessment and reappraisal techniques (36). The negative effect of high SMP on reassessment and reappraisal can be reduced by increasing PMP (47). In practice this can be done using breathing or mindfulness skills.

### Desensitization/Reconsolidation

**Figure 10:**
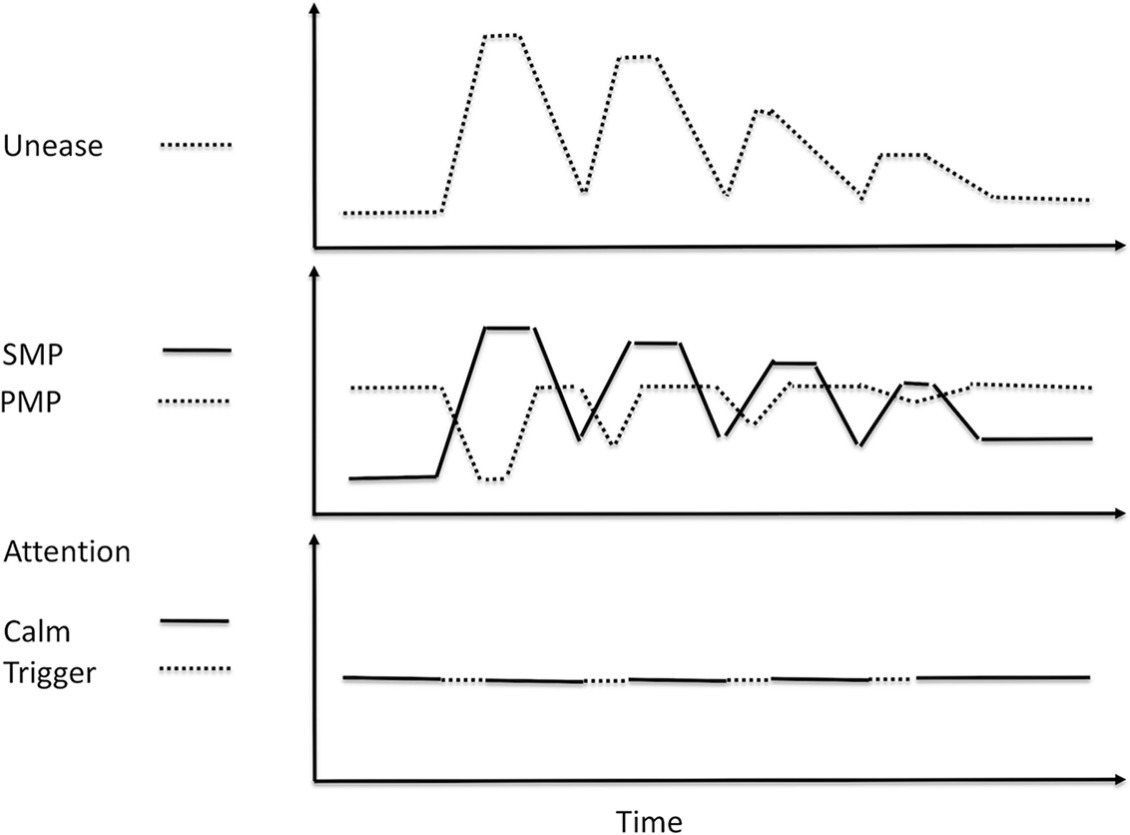
Desensitization. At first, the shift of attention to an unease-provoking trigger causes an increase in unease and SMP, and a decrease in PMP. As desensitization occurs, shifting attention to the trigger causes less of an increase in unease and SMP, but more importantly, the patient is able to maintain a higher level of PMP while placing attention on the trigger.

Desensitization and reconsolidation are well-known clinical techniques for reducing symptoms associated with stress. A PubMed search on these terms brings up over 1,500 citations. In the UM Model, desensitization and reconsolidation are techniques for reducing the level of unease associated with an experience. The experience may be a memory, a current situation, or an imagined future. In clinical practice, an effective desensitization process has the following steps (**Figure 10**):

1. The clinician and patient agree on a target experience to be desensitized.
2. The clinician helps the patient shift to a state of calm and maintain that for a minute or so.
3. The clinician helps the patient shift attention to the target experience in order for unease to increase.
4. The patient’s level of unease must increase to uncomfortable levels so the patient cannot reduce the unease without the help of the clinician. The time spent in this step varies, but in practice, only a minute or so is necessary.
5. The clinician aids patient to shift attention to a state of calm until the physical symptoms of unease subside to a tolerable level.
6. Steps 2 and 3 are repeated until the level of unease experienced in step 2 decreases to a level that does not cause avoidance or uncomfortable changes in SMP and PMP.

Research indicates various effective clinical techniques using the above process.

- Eye-movement desensitization and reprocessing (EMDR), extinction training (48).
- Pharmacologic interventions such as propranolol (49).

### Environmental Interventions

When demands are significantly greater than available resources, difficulty is high. The high difficulty will drive unease and SMP upward, despite individual’s attempts to adapt by selecting helpful responses to modulate unease, reduce SMP, and increase PMP. This requires clinical interventions to reduce difficulty by reducing the demands on the patient or increasing their resources.

## Clinical Applications of the Unease Modulation Model 

### Placebo Effect

The phenomenon occurs when patients enrolled in an intervention study experience benefit (e.g., drug effects) independent of the direct effects of the intervention. When patients experience negative effects, the term “nocebo” is used. The positive effects of placebos and the negative effects of noceboes can be explained in terms of the model.

- A person who receives a placebo experiences a reduction in unease. This decrease in unease causes a decrease in SMP and an increase in PMP, reducing physical and mental discomfort, and replenishing reserves. The negative effects of a nocebo are mediated by increases in unease, increases in SMP, and decreases in PMP, subsequently draining reserves (50).
- The change in unease associated with the placebo effect can influence behaviors as well. People may avoid making healthy changes because doing so causes high levels of unease. The placebo effect can reduce the unease associated with change and help patients to make healthy changes that then affect other components of the system (51).

The UM Model predicts that conditions strongly influenced by unease, and its associated autonomic effects, such as pain, will have a greater placebo response than conditions that are less influenced by unease, such as infection. The model also predicts that if a condition responds strongly to a placebo, then unease is likely to be a significant factor in the maintenance of that condition.

### Perfectionism

**Figure 11:**
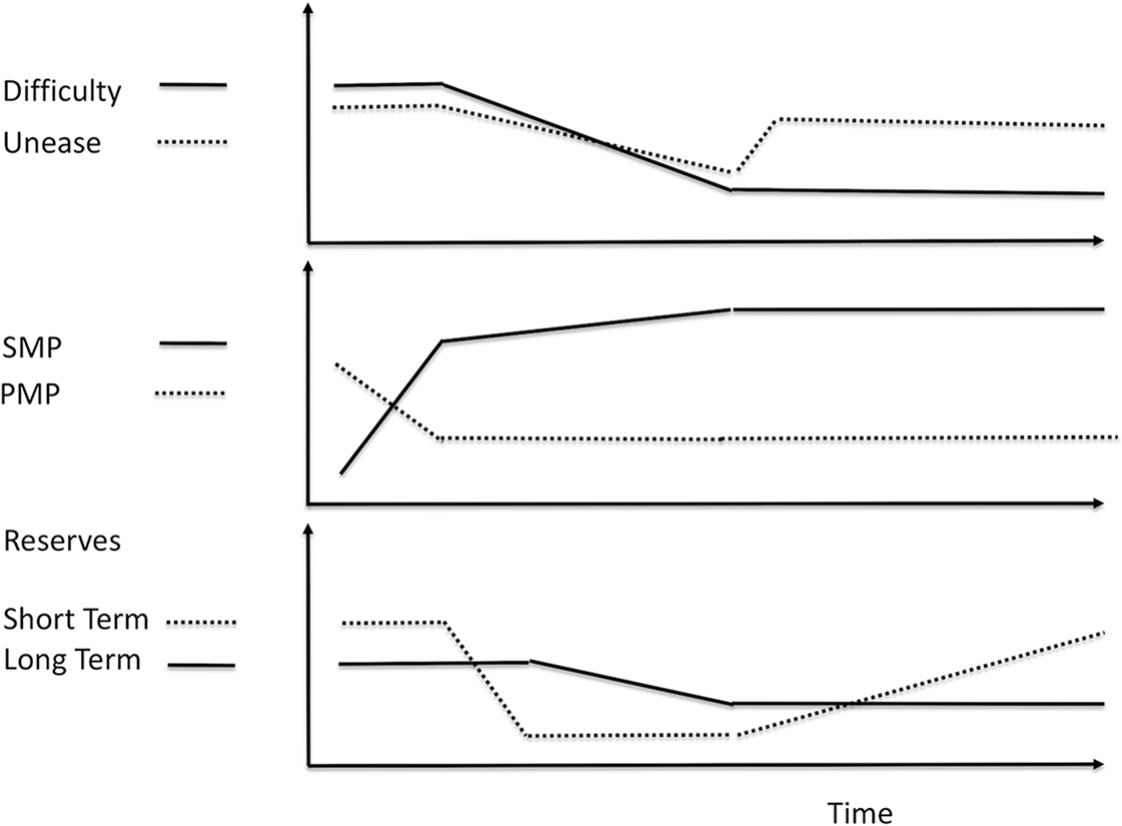
Perfectionistic response to difficulty. Perfectionism causes an increase in unease after difficulty decreases because the tasks were not done “right.” This eliminates the pleasure from doing the tasks. This increase in unease also keeps SMP elevated and keeps PMP reduced. The elevated SMP and reduced PMP slow the replenishment of reserves. While short-term reserves can be recharged eventually, the slow rate of replenishment may prevent long-term reserves from being recharged and they can become chronically depleted. This makes perfectionism a risk factor for a variety of conditions.

While not a diagnosis, perfectionism has a significant impact on many conditions by causing excessive unease. Ordinarily, a person will experience a pleasurable decrease in unease after completing a task. However, a person with maladaptive perfectionism will experience significant increases of unease after completing a task because they feel it was not done “perfectly.” The perfectionist is not able to reduce unease even by using relaxation exercises (52) (**Figure 11**). The chronic high unease elevates SMP, reduces PMP, and thus depletes reserves and inhibits effective responses.

- Reducing the perfectionism reduces the severity of the associated disorders (53). To reduce perfectionism, the person must learn to reappraise results so outcomes that are “good enough” reduce unease. Metacognitive skills for assessing the appraisal process and appraising the assessment process enable this.

### Anxiety Disorders

Anxiety disorders and post-traumatic stress disorder (PTSD) are characterized by high levels of unease, high SMP, and reduced PMP. The disorder is perpetuated by the selection of responses to avoid unease. The various types of anxiety disorder can be distinguished by what contributes to the elevations in unease (e.g., spider phobia). Treatment involves reducing the unease and avoidance, correcting inaccuracies in assessment and appraisal, and training fewer avoidant responses. Various methods can be used to attain these goals (see **Table 3**).

**Table 3:** Common applications of Unease Modulation Model (UM Model) to anxiety disorders.

Disorder	Dysregulation	Treatment methods
Phobia	Excessive unease in presence of feared stimulus	Desensitization to feared stimulus
Panic disorder	Excessive unease associated with symptoms of elevated SMP	Desensitization to sensations of elevated SMP
Performance anxiety	Excessive unease about audience response	Desensitization to various feared audience responses
	Unease about not being flawless (perfectionism)	Reappraisal skills
	Reduction in fine-motor or cognitive skills when SMP is high	Overtraining the skills so reduction does not impair performance; increasing PMP when unease is high
Social anxiety	Excessive unease about rejection	Desensitization to being rejected
	Unease about having to perform effectively if accepted	Social skills training
Generalized anxiety	Excessive unease about unease	Desensitization to being uneasy “It’s safe to feel fear”
PTSD	High baseline SMP and low baseline PMP	Relaxation training, mindfulness
	Unease increases rapidly from traumatic memories	Reconsolidation
	Current stimuli cause conditioned increases in unease and SMP	Desensitization
	Inaccurate appraisal causing high unease in safe situations	Reappraisal skills
	Assessed difficulty greater than environmental difficulty	Reassessment skills

SMP, sympathetic nervous system activation; PMP, parasympathetic nervous system activation.

### Depression and Burnout

Depression and burnout are conceptualized as arising from depleted long-term reserves. When long-term reserves are depleted, SMP remains chronically elevated to meet demands, even when difficulty is low. High SMP is experienced as pushing, and so for the depressed patient, everything feels like an effort.

- High SMP tends to increase unease and the increased unease reduces PMP, making it difficult to replenish reserves.
- Depleted reserves impair the ability to complete tasks and experience resulting reductions in difficulty and unease.
- Inability to reduce unease leads to a lack of pleasure, and even if a task is completed, the elevated SMP keeps unease high and pleasure is not experienced.

These feedback loops reinforce each other and perpetuate the condition. Treatment must address multiple components and interactions while accommodating the fact that the patient’s resources are depleted and thus their energy available to make changes is limited.

The initial focus of treatment must be on replenishing reserves by increasing PMP and reducing SMP and unease. The high level of unease in such patients can make it difficult for them to apply techniques to replenish reserves successfully. As long-term reserves increase, the causes of depletion must be addressed to prevent a rapid recurrence.

One cause of depletion is excessive demands, which can be common in patients who have to meet both family and work obligations. Another is consistently high unease that may be due to abuse, deprivation, distress in a primary relationship, or having to violate core values in order to maintain a work or living situation. Addressing these issues often requires the patient to make significant changes in assessment, appraisal, and response selection processes, and the patient may also require assistance in changing their environment.

### Chronic Pain

Patients with chronic pain report unease when their pain increases and increases in pain when unease increases. A variety of approaches can be used to reduce the patient’s unease, tailored to their needs (**Table 4**).

**Table 4:** Examples of methods for treating chronic pain based on UM Model.

Disorder	Dysregulation	Treatment methods
Chronic pain	Excessive attention on pain vs. other sources of pleasure	Environmental changes to create opportunities for succeeding at challenges
	Excessive unease about pain	Reappraisal methods; desensitization methods
	Exaggeration of demands and underestimation of resources	Reassessment methods
	Pain sensations	Imagery, hypnosis/self-hypnosis
	Reduced PMP when in pain	Mindfulness

Interestingly, patients will describe less pain if they experience a level of difficulty that does not cause high unease, i.e., a challenge. The increased SMP required to deal with the difficulty helps them focus their attention on meeting the difficulty and that reduces their experience of pain. Since patients who suffer from chronic pain often have reduced physical resources, their ability to succeed at meaningful challenges is compromised and they may need help in finding ones they can succeed at. If they live in an impoverished setting, then they may need assistance to be able to have the opportunity to engage in challenging activities.

Note that opioids reduce unease significantly and quickly. Therefore, opioids are perceived to reduce pain largely through their effect on unease. However, opioids do not change the other components of the system that are involved in chronic pain. If those components are not changed, then the opioids will not be helpful. Worse, the patient may use the opioids inappropriately to relieve other sources of unease. Finally, the opioids may induce changes in the brain that cause the patient to experience unease simply because of the absence of the opioid. When this occurs, the patient has an addiction to opioids. This is explained in more detail in the supplement.

### Addiction

An addictive behavior has two necessary and sufficient conditions:

1. The behavior reduces unease but increases difficulty—i.e., it is an unhelpful habit
2. The absence of the behavior increases unease that is only fully reduced by the behavior.

The second condition is what separates an addiction from unhealthy habits. Many behaviors that satisfy the first condition, i.e., that reduce unease and increase difficulty, even behaviors involving substance use, are not necessarily addictions. For example:

- Procrastination is rarely an addiction as people do not generally stop an activity in order to procrastinate.
- Anger outbursts are also not usually addictive as people who have anger outbursts rarely find the absence of anger something to be uneasy about.
- Someone who drinks alcohol excessively at parties to reduce social anxiety may not have an addiction to alcohol. If the absence of alcohol does not cause unease except in social situations, then the person has a dangerous habit, but does not have an addiction. In clinical practice, if such a person receives effective treatment for the social anxiety, then the unhealthy alcohol use stops.
- The vast majority of soldiers who used heroin in Vietnam stopped using heroin when they returned to the United States without needing any treatment (54).

A person with an unhelpful habit experiences unease from general sources, “general unease,” and must learn to cope with that unease without engaging in the unhelpful habit. The person who has an addiction to using a drug or has another addictive behavior has a much more complicated problem. The addict experiences two types of unease, “general unease,” and unease from the absence of the addictive behavior, “absence unease.” This means the addict is often experiencing significantly higher levels of unease than a person who is not an addict. Since high levels of unease have detrimental effects on SMP and PMP, the addict experiences negative effects from those more often. The addict may also have a hard time determining the source of their unease and thus choose responses that reduce “general unease” when the source is “absence unease.” For example, the addict may attribute the source of their unease as relationship distress when its main source is the absence of the addictive behavior.

To recover, addicts must learn two sets of skills.

- The first is to experience “general unease” without engaging in the addictive behavior.
- The second is to experience “absence unease” without engaging in the addictive behavior.

The first set of skills includes methods that help people who are not addicts deal with unease. The second set of skills is specific to treating addiction. In addition to learning both sets of skills, the addict must also learn to appraise the source of their unease accurately, as applying a technique to reduce general unease will not reduce “absence unease” and vice versa.

One method of treating the “absence unease” is to apply the method of desensitization (listed in Desensitization/Reconsolidation). The addict is presented stimuli that trigger “absence unease” and taught techniques to attenuate it without engaging in the behavior. This is risky because if the “absence unease” is not attenuated it could lead to use of the behavior. In practice, the “absence unease” can be triggered in a setting in which the behavior is not possible, such as an office visit or treatment setting.

## Social and Organizational Implications of the Unease Modulation Model

The model for stress has implications for organizations and groups that arise from the responses of the individual members of these organizations and groups.

### Health Advertising

Advertising works by causing people to experience unease while offering a product or service to reduce that unease. The images, music, words, volume, pace, and tone of speech in advertisements are all deliberately chosen to manipulate unease in the viewer so that the offered product will be associated with a large reduction in unease. Advertising is not done to inform, unless the information presented is in the service of manipulating the unease. Advertising about health is no exception; ads for health-related products are increasing unease in the population. However, increased unease contributes to the development and exacerbation of stress-related disorders, and so, advertising for health-related products may be making people sicker.

### Abusive Relationships

A person who is being abused in a relationship will often plan to leave the relationship but then decide to stay in it when the abuser apologizes, even though they know that the abuse will reoccur. The person is usually frustrated by their behavior and feels quite negative about themselves. This pattern of wanting to leave but deciding to stay comes from the fact that behaviors that reduce unease are reinforced. After an episode of abuse, when the person is considering leaving, unease is high. When the abuser apologizes and the person agrees to stay because things are better, unease drops quickly. Now, the idea of leaving causes a large increase in unease and is aversive. Thus, staying in the abusive relationship becomes the learned behavior. This is simply a dysfunctional outcome of a natural learning process and not something inherently pathological in the abused person. When patients are shown how they are training themselves to tolerate abuse, they seem to be able to stop blaming themselves and become more capable of taking actions to change the relationship.

### Marital Distress

When one partner in a relationship is uneasy, the other tends to become uneasy (55, 56). The more important the relationship, the more uneasy each partner feels. This phenomenon can be called negative resonance. Just as increases in unease can resonate in a couple, decreases in unease can resonate as well. This is called positive resonance.

Positive resonance, when both partners experience reductions in unease, is pleasurable. Negative resonance is aversive. One principle in treating relationship distress is for couples to learn to enhance positive resonance and inhibit negative resonance. Partners can reduce negative resonance by learning to increase PMP when their partner is uneasy. That enables them to stay aware of and engaged with their partner’s unease without increasing their own unease or their partner’s.

This is especially useful when the couple is discussing a difficult topic that is also emotionally charged. Thus, the UM Model predicts that during a discussion, the unease in each partner will tend to resonate, increasing the unease of each and reducing their cognitive and social skills. As the unease escalates, the discussion will become less and less helpful and more and more damaging. However, if each partner becomes skilled at increasing PMP as unease escalates, then the negative resonance and the impairment of cognitive skills are less likely, and the discussion is more likely to be helpful.

### Organizational Defensive Routines

When people in organizations discuss issues that do not increase unease, effective discussion is achieved (i.e., difficulty is reduced). However, if the issue is emotionally charged (i.e., the participants are uneasy about the topic and uneasy about evoking unease in each other), discussions are likely to be ineffective (57).

- The UM Model suggests that ineffective discussions are driven largely by the increases in SMP and decreases in PMP associated with the increases in unease.
- These changes in SMP and PMP cause reductions in cognitive, communication, and social skills.
- The decreasing skills make selecting responses to reduce difficulty much more challenging, and so responses to reduce unease are selected instead (e.g., end the meeting early without a resolution, pretend to agree while harboring private reservations).

The solution is to have the participants focus on improving cognitive skills such as listening skills or nonviolent communication skills. However, the UM Model predicts that unless the participants are able to modulate their physiology by increasing PMP when unease is high, the enhanced cognitive skills will continue to be used in the service of reducing unease rather than difficulty. The goal is to increase PMP and thus reduce the negative effects of high SMP on cognitive and social skills.

### Clinging to False Beliefs

“It is difficult to get a man to understand something, when his salary depends upon his not understanding it.”—*Upton Sinclair*

An interesting phenomenon is that people of all intelligence levels will tend to adhere to a preexisting belief about an emotionally charged issue rather than to accept a new belief that is more accurate.

- The more people are confronted with the evidence against their belief, the more they tend to adhere to it.

This is consistent with the UM Model. Uncertainty tends to increase unease. If people are already uneasy about an issue, then increased uncertainty will be even more aversive. In order to avoid increasing uncertainty, they will tend to avoid considering the more accurate belief, as doing so will require them to experience the uncertainty of letting go of their belief. The avoidance may be so severe that they will literally not be able to see the evidence against their belief.

The UM Model further suggests that if we want someone to consider a belief they would generally reject, we need to make sure we present the alternative belief in a manner that does not increase unease more than the person can tolerate. Since increased PMP reduces unease, then the presentation of the new belief in an environment that increases PMP increases the likelihood that the new belief will be considered (58). This may be a mechanism responsible for the observation that pharmaceutical industry-sponsored meals to physicians contribute to increased prescribing (59).

### Unease and Populism or Radicalization

A sense of belonging to a group or being part of a tribe, a strong group affiliation, has a powerful effect on reducing unease. A sense of belonging can protect against unease from other sources such as threat, pain or illness, or high levels of difficulty.

- If people can be made to feel uneasy and offered a strong group affiliation as a solution, the reduction in unease is pleasurable and the group affiliation is reinforced.
- If the people in the group can also be made to fear people who are outside the group, then the reduction in unease from belonging to the group can now be self-reinforcing.

Demagogues therefore manipulate unease in their adherents in order to create a polarization of their adherents as a group opposed to others. Since reductions in unease influence perception, the members of the group may become unable to see how those outside of the group are being falsely represented. They are literally blinded by the reduction in unease that comes from participating in the group.

As pointed out earlier, belief reduces uncertainty, which reduces unease. The members of a community based on shared belief experience reduced unease both from the shared belief and from the group bonding. This makes group members tend to constrain not only their behavior but also their thinking as any challenge to the shared belief will increase unease from both the questioning of the belief and the risk of losing group membership. The reduction in unease from adhering to group values and behavioral norms can be so large that it causes people to violate previously held values and commit atrocities that previously would have caused intolerable levels of unease.

### Public Health

The association between poverty, oppression, and stress-related health conditions has been documented (60). Often, this is presumed to come from the negative effects of meeting demands with limited resources, i.e., high difficulty. However, chronically high unease may have a more pervasive and more negative effect. External influences, such as threat, marginalization, racism, profiling, and the like, can all increase unease independent of difficulty.

- People who experience negative social forces, and associated chronic unease, suffer from negative health effects caused by increased SMP and suppressed PMP and depleted reserves.
- Chronic high unease is unhealthy, and it causes people to select responses to reduce it. If the environment does not provide opportunities for helpful responses to reduce unease, then unhelpful responses such as substance use or behavioral disorders are more likely to be selected instead.

The UM Model predicts that reducing difficulty alone will not reduce stress-related conditions if unease remains high. Introducing distractions such as entertainment or mindfulness will not lead to long-term reductions in unease or stress-related health conditions. The environmental difficulties and external sources of unease must be mitigated by social change and governmental interventions in order to obtain effective long-term improvements in health (61).

## Conclusion

What are colloquially referred to as “stress” and the “effects of stress” can be explained using a model that breaks stress down into phenomenological components, i.e., difficulty, unease, SMP, PMP, and reserves, with unease having particular importance.

People who experience stress can be taught to identify and distinguish the components and the interactions among them. Subsequently, they can be taught how to modulate the components, unease in particular, to improve their health, relationships with others, and effectiveness at reducing demands in a satisfying and sustainable manner.

The benefits of many therapeutic modalities that are used to treat stress-related conditions can be explained by their influence on the components and interactions outlined in the UM Model. When people understand how the suggested interventions are supposed to affect the various components, they are more likely to apply the interventions successfully.

The UM Model also suggests how some social or organizational problems arise and how various methods may affect those problems in helpful or unhelpful ways. In particular, it warns against the tendency of humans to allow unease to drive perception, assessment, and response selection and offers methods that reduce this tendency by modulating physiologic as well as cognitive processes.

The UM Model is not meant to explain all aspects of human behavior or internal experience. For example, it has not been applied to patients with psychotic disorders such as schizophrenia and schizoaffective disorder or patients who are in a full manic episode. It especially is not meant to imply that one control variable can be defined and used to predict mental and behavioral functioning. Close work with patients over many years gives numerous counterexamples of any mental “theory of everything.” The strength of the model is that it provides an easily understood guide for many patients. For those who do not find it useful, it is to be discarded and some other model used instead. For clinicians, the model can be helpful by giving a framework for understanding how to apply techniques from a wide variety of psychotherapeutic modalities, but it is not meant as a substitute for those modalities. For researchers, the model may be tested in the development of interventions. For the layperson, the UM Model may be applied to make tangible change in their lives.

## Author Contributions

JA developed the UM Model and its applied and clinical applications. JPA contributed to the application of the model in a nonclinical setting while collecting data with law enforcement professionals. JA wrote the first draft of the manuscript; JPA wrote sections of the manuscript. All authors contributed to manuscript revision and read and approved the submitted version.

## Funding

JPA was funded by grant from the Government of Ontario, Ministry of Labour (ROP 15-R-021) to conduct research related to police as described in this article. However, the Ministry had no other involvement in the conceptualization, design, analysis, decision to publish, or preparation of this manuscript.

## Conflict of Interest Statement

The authors declare that the research was conducted in the absence of any commercial or financial relationships that could be construed as a potential conflict of interest.

## Supplementary Material

Figure S-1 Environment – Experience – Response. The process of perception gives rise to our experience based on our environment. The process of selection gives rise to our response based on our experience. The process of influence gives rise to a change in the environment based on our response. Perception, selection, and influence are each conditional probability distributions.

Figure S-2 The UM-Model. Unease has more influence on experience and response than does difficulty. Increases in unease can increase or decrease difficulty. Increases in unease cause larger increases in SMP than difficulty. Increases in unease cause decreases in PMP, and increases in unease can cause further increases in unease. Experience has an increasing or decreasing effect on unease. Increases in SMP will also increase or decrease unease. Increases in difficulty tend to increase unease. Increases in PMP will decrease unease. Note that unease is affected by and effects more components than any other variable. This gives unease a much greater level of influence on the evolution of experience and response.

Figure S-3 Legitimate use of opioid pain medication reduces unease from other sources. A and B – Pain is high and the patient takes a prescribed opioid to reduce pain. The opioid wears off and the pain increases. A – The opioid is taken when pain is high and unease from sources other than pain is low. Taking the opioid does not reinforce use of the opioid to reduce unease. B – Unease from other sources other than pain is high as will occur from time to time. Taking the opioid reduces unease from sources other than pain. The reduction in unease after taking the opioid reinforces the use of the opioid to reduce unease. This happens even if the patient has no intention of using the opioid to reduce unease from other sources.

Figure S-4 Legitimate use of opioid pain medication alters perception of pain so unease is perceived as pain. A and B – The patient perceives pain as high and the patient takes a prescribed opioid to reduce pain. The opioid wears off and the pain increases. A – The opioid is taken when pain is high and unease from sources other than pain is low. Taking the opioid does not reinforce use of the opioid to reduce unease. B – Unease from other sources other than pain happens to be high as will occur from time to time. This is perceived as pain. Taking the opioid reduces the unease and the patient perceives a reduction in pain. The patient is not aware that their perceptual process has changed, and this occurs in spite of the patient’s intention to use the opioid solely to reduce pain. Nor would a prescriber be able to determine the difference. At this point the patient still does not experience unease simply from not taking the opioid, i.e. does not experience "absence unease".

Figure S-5 Legitimate use of opioid pain medication reinforces use to reduce "absence unease." A – As tolerance to the opioid develops the patient experiences high unease as the opioid wears off. This is absence unease. The unease is perceived as pain and the patient is using the medication as instructed and reporting honestly to the prescriber. However, the use of the opioid to reduce absence unease is being reinforced, and the patient is developing an opioid use disorder that is likely to worsen with continued use. Note that neither the patient nor the prescriber will realize this as the patient is continuing to use the medication as prescribed. The key warning sign is that the patient is tending to use the opioid with increasing temporal regularity.

Figure S-6 Early opioid use disorder when using prescribed opioids analgesics. A – The opioid is taken when absence unease is high. The patient is developing tolerance to the opioid so absence unease is occurring more quickly after taking the opioid. In the absence of any exacerbation in the patient’s medical condition that was causing the pain, this is strong indication that the patient is using the opioid to reduce absence unease that is experienced as pain. Note, the patient is experiencing “real pain” and is being honest in their reporting. In clinical practice the increased frequency of use is not as great as is illustrated in the figure. The increase in frequency depicted is exaggerated for illustration purposes.

Figure S-7 Early preventative measures reduce the development of opioid use disorder from prescribed opioid analgesics. A – The opioid is taken when pain is high and unease from sources other than pain is low. Taking the opioid does not reinforce use of the opioid to reduce unease. B – Unease from other sources other than pain happens to be high as will occur from time to time. C – Use of non-opioid interventions to reduce pain is associated with the reduction in unease from other sources. This increases the ability of the non-opioid intervention to reduce both pain and unease. Interventions of type C will be most effective in preventing opioid use disorder if they are initiated as soon as possible after the initiation of opioid analgesia and if they are done consistently whenever the opioid analgesic is taken, and more frequently if possible.
