# Supplementary figures and images for: The Unease Modulation Model: An Experiential Model of Stress With Implications for Health, Stress Management, and Public Policy
Source: Front Psychiatry. 2019 Jun 7;10:379. doi: 10.3389/fpsyt.2019.00379 (PMC6567485; doi:10.3389/fpsyt.2019.00379)

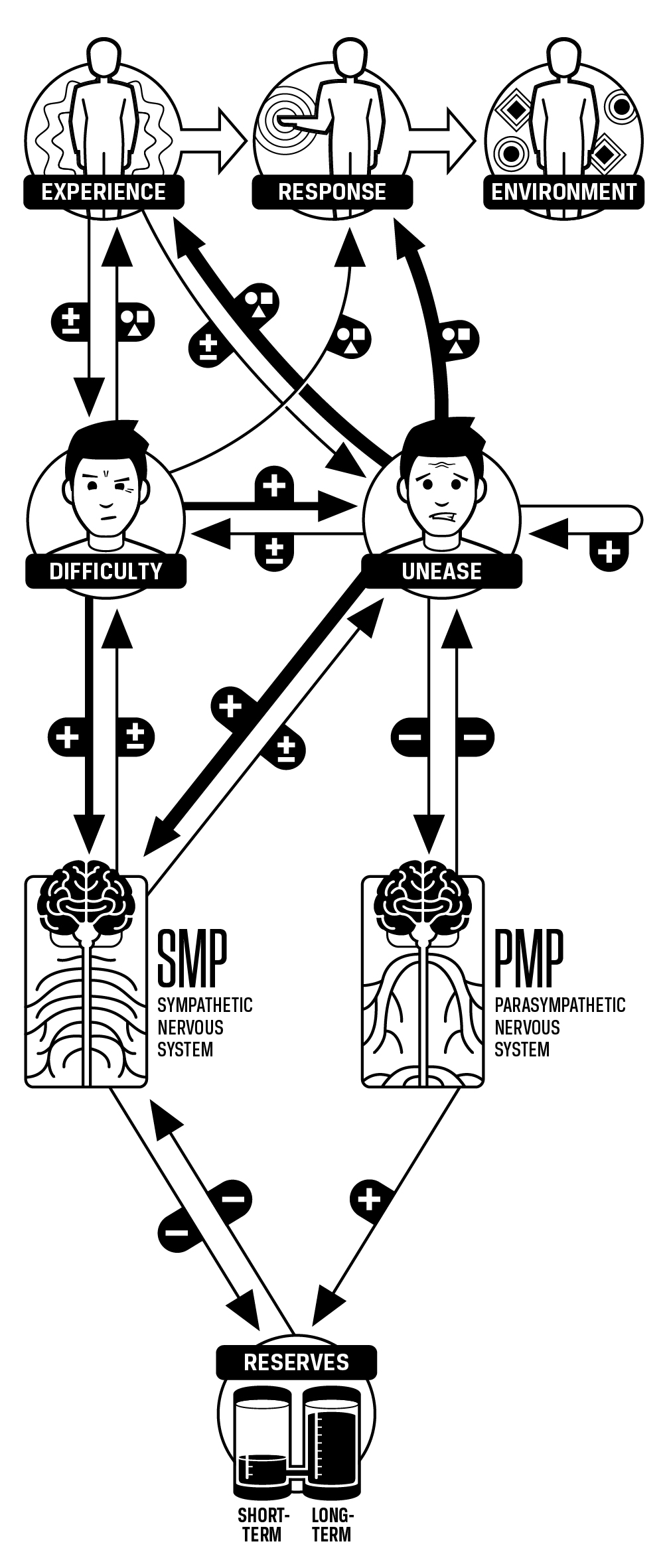

Supplement: Figure S-1 — Environment – Experience – Response. The process of perception gives rise to our experience based on our environment. The process of selection gives rise to our response based on our experience. The process of influence gives rise to a change in the environment based on our response. Perception, selection, and influence are each conditional probability distributions. [file DataSheet_1.zip › Figure S-2 Unease Modulation Model.jpg]

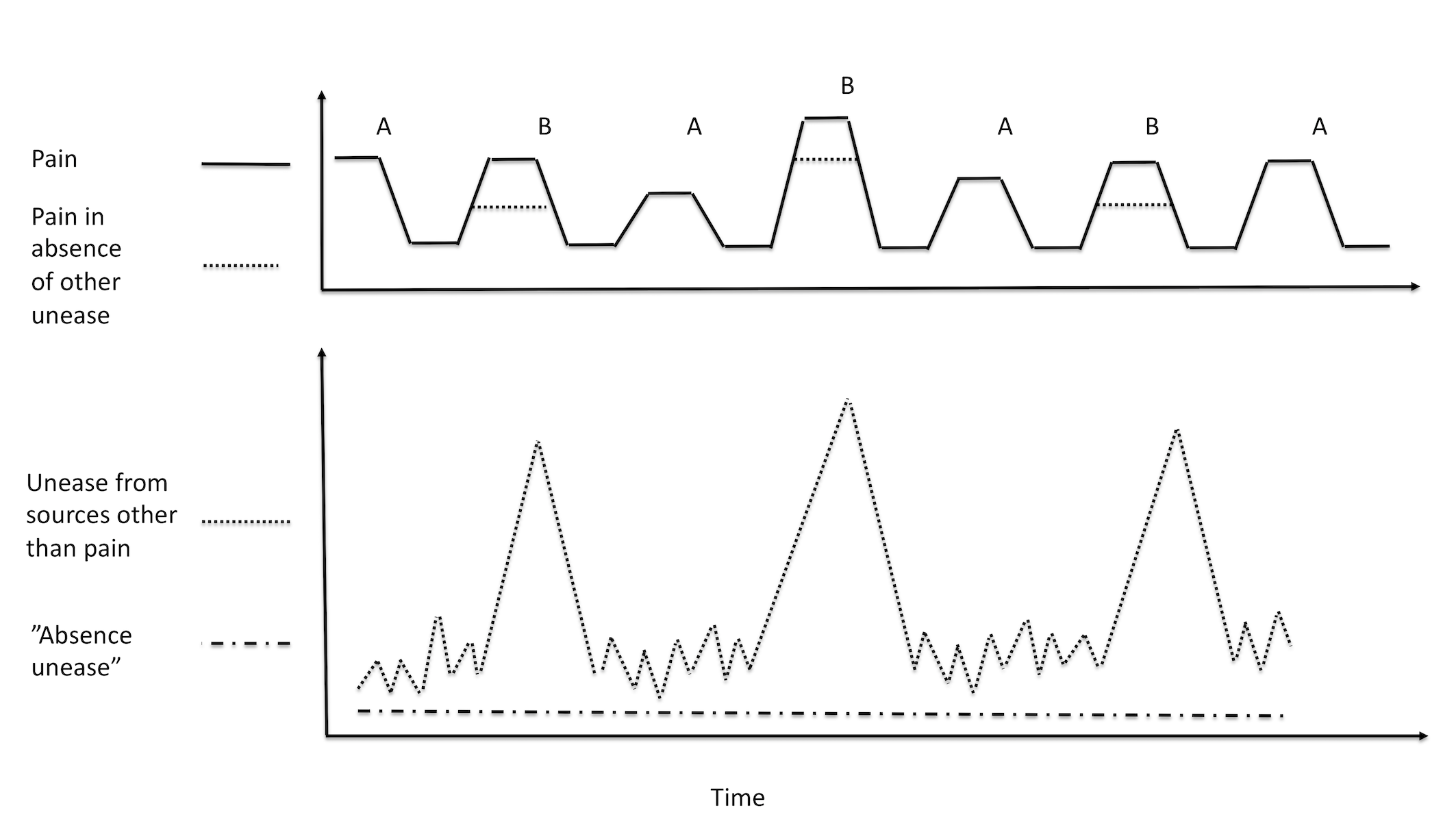

Supplement: Figure S-1 — Environment – Experience – Response. The process of perception gives rise to our experience based on our environment. The process of selection gives rise to our response based on our experience. The process of influence gives rise to a change in the environment based on our response. Perception, selection, and influence are each conditional probability distributions. [file DataSheet_1.zip › Figure S-4 Legitimate use of opioid pain medication alters perception of pain so unease is perceived as pain LZW.tiff]

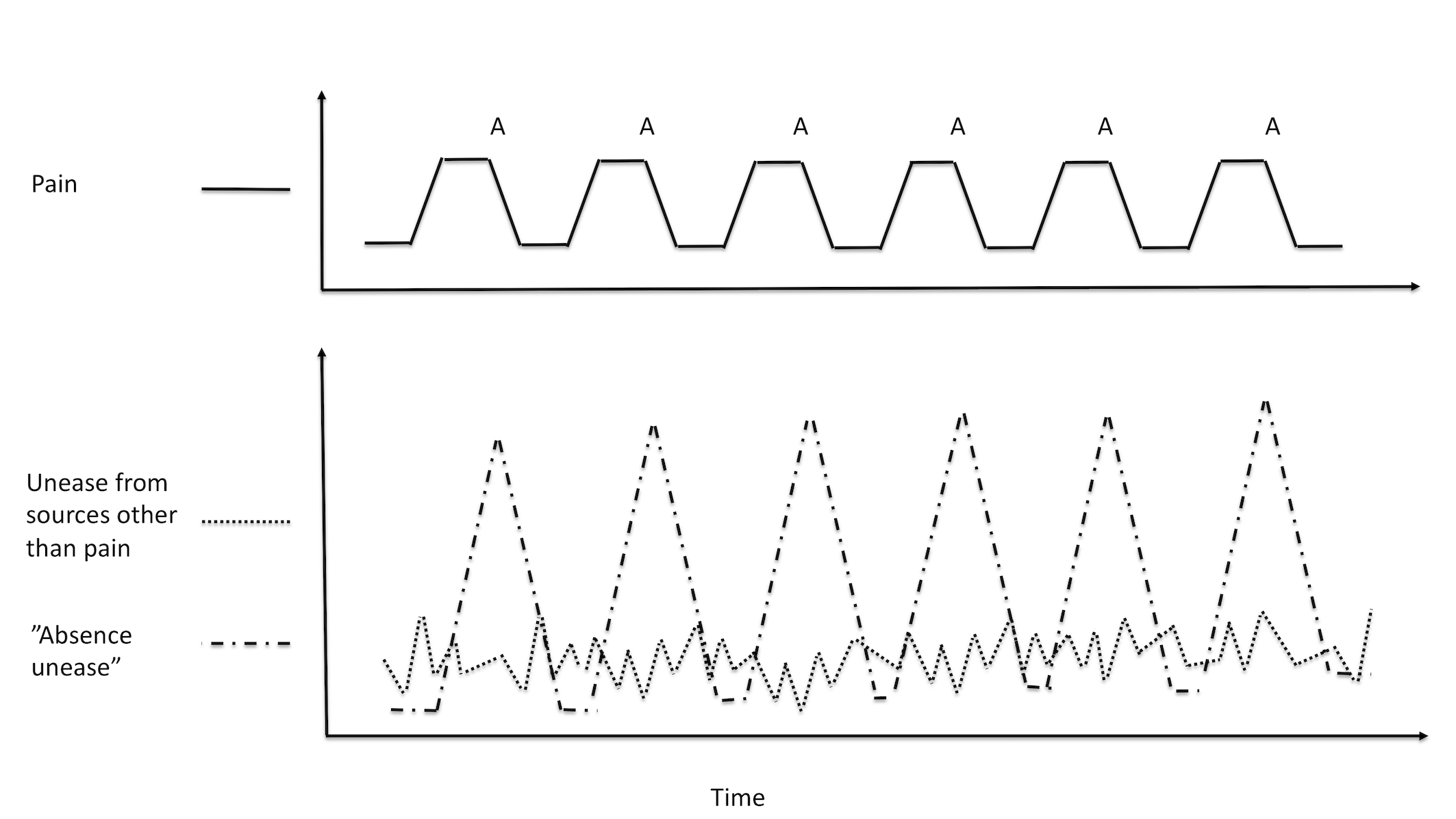

Supplement: Figure S-1 — Environment – Experience – Response. The process of perception gives rise to our experience based on our environment. The process of selection gives rise to our response based on our experience. The process of influence gives rise to a change in the environment based on our response. Perception, selection, and influence are each conditional probability distributions. [file DataSheet_1.zip › Figure S-5 Legitimate use of opioid pain medication reinforces use to reduce "absence unease" LZW.tiff]

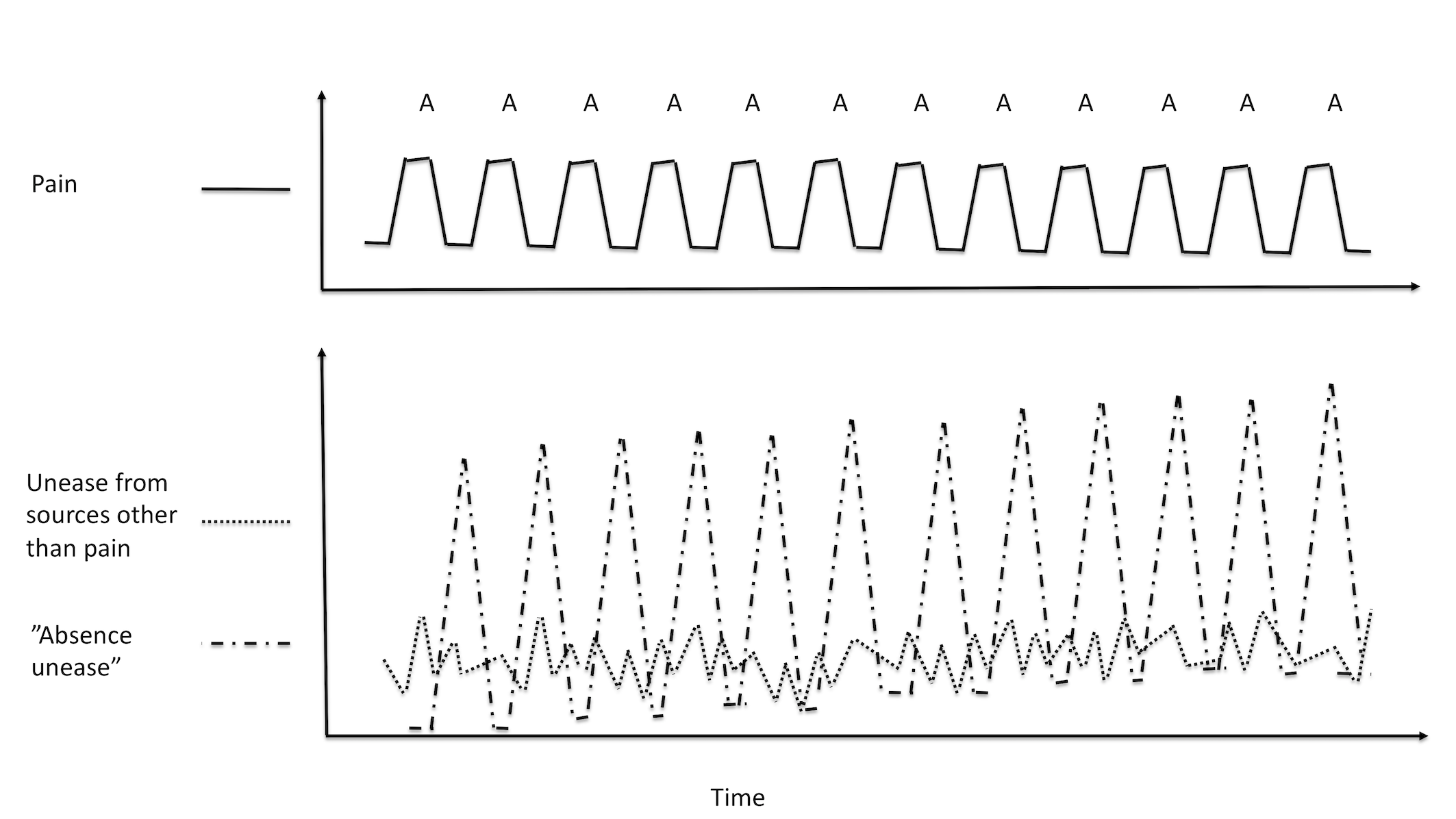

Supplement: Figure S-1 — Environment – Experience – Response. The process of perception gives rise to our experience based on our environment. The process of selection gives rise to our response based on our experience. The process of influence gives rise to a change in the environment based on our response. Perception, selection, and influence are each conditional probability distributions. [file DataSheet_1.zip › Figure S-6 Early opioid use disorder when using prescribed opioid analgesics LZW.tiff]

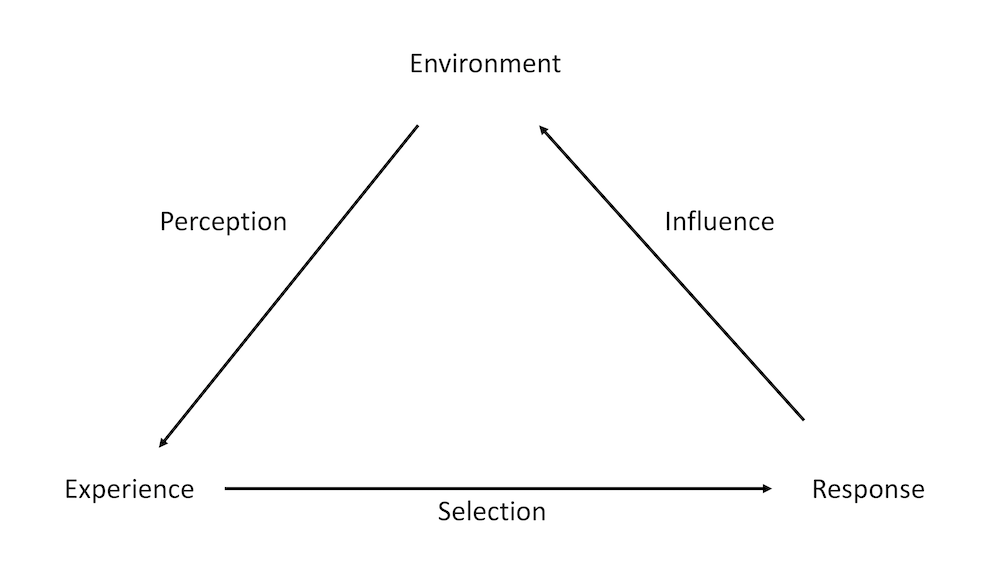

Supplement: Figure S-1 — Environment – Experience – Response. The process of perception gives rise to our experience based on our environment. The process of selection gives rise to our response based on our experience. The process of influence gives rise to a change in the environment based on our response. Perception, selection, and influence are each conditional probability distributions. [file DataSheet_1.zip › Figure S-1 Environment - Experience - Response LZW.tiff]

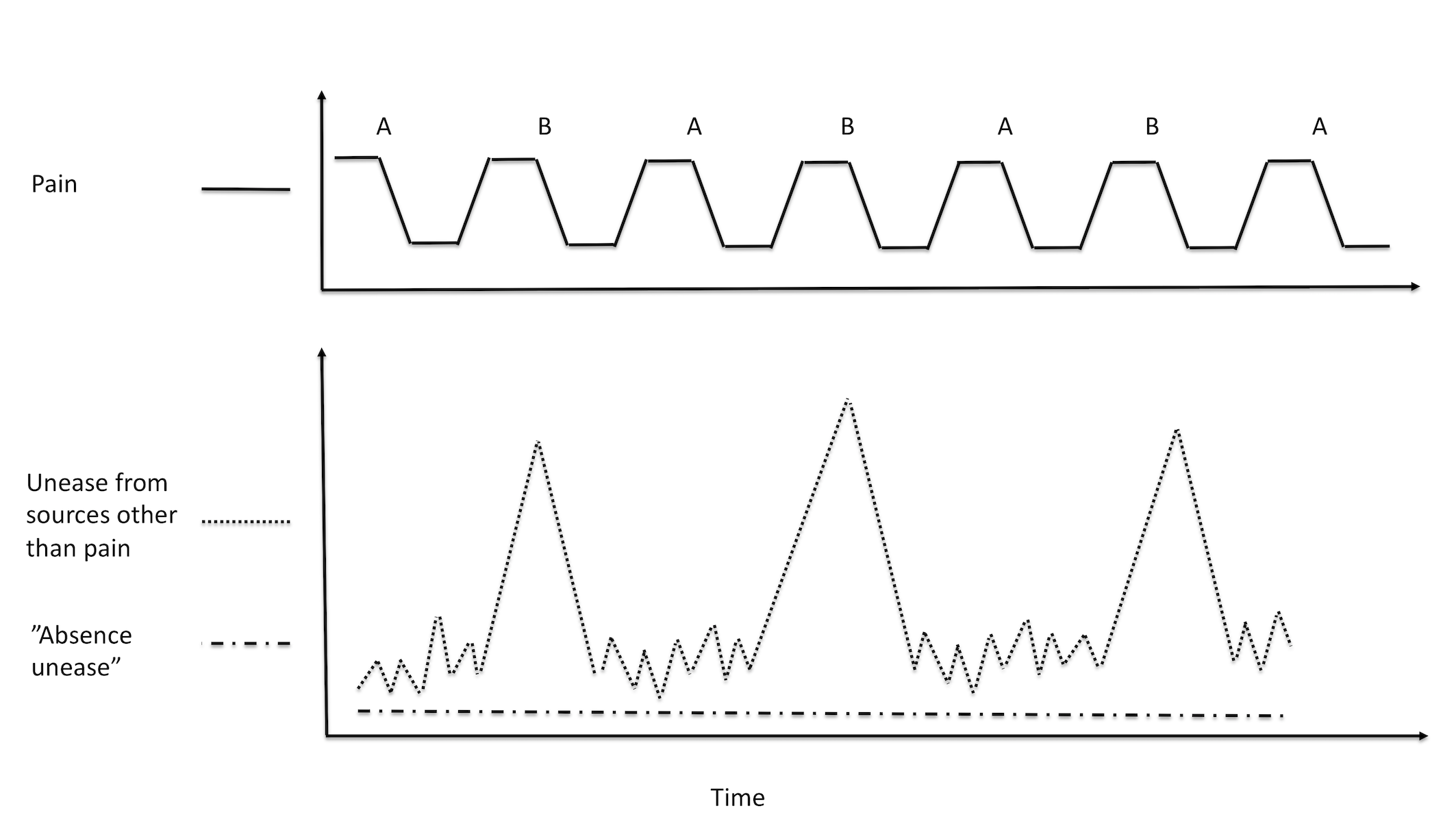

Supplement: Figure S-1 — Environment – Experience – Response. The process of perception gives rise to our experience based on our environment. The process of selection gives rise to our response based on our experience. The process of influence gives rise to a change in the environment based on our response. Perception, selection, and influence are each conditional probability distributions. [file DataSheet_1.zip › Figure S-3 Legitimate use of opioid pain medication reduces unease from other sources LZW.tiff]

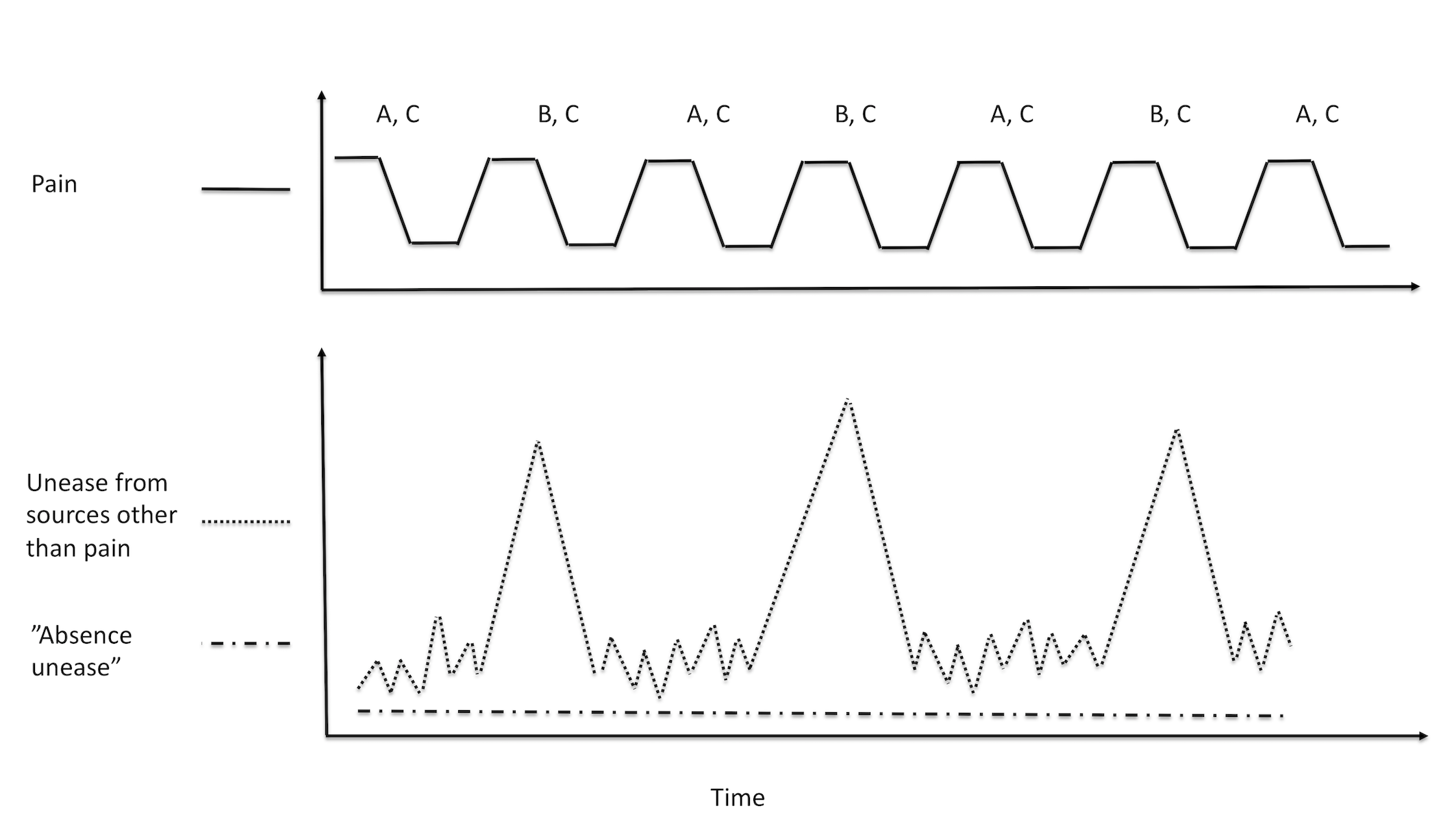

Supplement: Figure S-1 — Environment – Experience – Response. The process of perception gives rise to our experience based on our environment. The process of selection gives rise to our response based on our experience. The process of influence gives rise to a change in the environment based on our response. Perception, selection, and influence are each conditional probability distributions. [file DataSheet_1.zip › Figure S-7 Early intervention measures reduce the development of opioid use disorder from prescribed opioid analgesics LZW.tiff]
