# Supplementary material for: The Unease Modulation Model: An Experiential Model of Stress With Implications for Health, Stress Management, and Public Policy
Source: Front Psychiatry. 2019 Jun 7;10:379. doi: 10.3389/fpsyt.2019.00379 (PMC6567485; doi:10.3389/fpsyt.2019.00379)
Supplement: Supplementary file 2 [file DataSheet_2.docx]

# Supplement to the Unease-Modulation Model: A mathematical formulation and application to opioid-dependence arising from use of opioid analgesics.

Theories of perception and consciousness use conditional probability distributions to structure the interactions between the world, or environment, experience, and response (Fields, et. al. 2017; Hoffman and Prakash, 2014). The starting point for the Unease Modulation Model (UM-Model) are the processes that give rise to experience and response from our environment.

The probability of an experience, prob (Exp), arises from the perceptual process operating on a particular environment (Env). The probability of a response, prob (R), arises from the selection process operating on a particular experience (Exp). The probability of a particular new environment, prob (Env), arises from the influence process operating on a particular response, see Supplementary Figure S-1.

prob ( Exp ) = Perception ( Env )

prob ( R ) = Selection ( Exp )

prob ( Env ) = Influence ( R )

The possible environments, experiences, and responses are too complex to be described numerically and stress has been used as a non-negative numerical variable that can be used to reduce uncertainty about the chance of particular experiences or responses occurring. We would state "The probability of a particular experience arises from the perceptual process operating on a particular environment given a particular level of stress," and "The probability of a particular response arises from the selection process operating on a particular experience given a particular level of stress." Note that stress is not expected to affect the way a response changes the environment. To denote this, the equations can be written:

prob ( Exp ) = Perception ( Env | Stress )

prob ( R ) = Selection ( Exp | Stress )

prob ( Env ) = Influence ( R ).

The goal of using stress is by knowing a person's level of stress, to at least some precision, one can predict a person's experience and response more accurately. However, while people often describe high levels of stress as having harmful effects on their experience and response, the same people also describe situations in which high levels of stress have beneficial effects on their experience and response. If the same level of stress can cause opposite effects, then stress is not as useful for predicting experiences and responses.

One solution would be to redefine stress as something other than a single number. The UM-Model applies this idea in redefining stress. Specifically stress is defined as a vector of 5 non-negative numbers, Difficulty (Diff) , Unease (U) , Sympathetic Nervous System (SMP), Parasympathetic Nervous System (PMP), and Reserves (V).

Experience and response are both affected by these 5 components. The probability of a particular experience comes from the perceptual process operating on the current environment given the current levels of difficulty, unease, SMP, PMP, and reserves. The probability of a particular response comes from the selection process operating on the current experience given the current levels of difficulty, unease, SMP, PMP, and reserves.

prob ( Exp ) = Perception ( Env | Diff, U, SMP, PMP, V )

prob ( Response ) = Selection ( Exp | Diff, U, SMP, PMP, V )

In practice we want to be able to estimate the probability that an experience will have a particular quality, say anxiety, or that a response will include a certain action, say avoidance. In order to work with the probabilities some notation is necessary and the following notations are introduced.

First, a sequence of symbols is defined to denote how much the person's environment, experience or response consists of a particular element. The sequence of symbols is:

A ∅ "B" means A contains no presence of the element B,

A ⊃ "B" means A contains element B as well as other elements,

A ~ "B" means A contains element B and other elements are not significant.

For example,

Exp ∅ "anxiety" means the person in experiencing absolutely no anxiety,

Exp ⊃ "anxiety" means the person is experiencing anxiety but is perceiving other elements of experience as well,

Exp ~ "anxiety" means the person is experiencing anxiety and other elements of experience are not significant.

Note that the symbols refer to the pervasiveness of the element, not the intensity of the element, though intensity and pervasiveness are likely to be correlated.

Second, the components, difficulty, unease, SMP, PMP, and reserves are numerical, but precise numbers can be difficult to assign to these. However, the qualitative values, none, low, moderate, and high can be used as these are what people describe subjectively.

If a person describes difficulty as high and unease as low, we would write,

Diff ~ "high" and U ~ "low".

Third, the notation "+" and "-" will be used to describe the influence a component has on the probability of an event. A "+" will mean an increase in the component increases the probability of an event. A "-" will mean an increase in the component decreases the probability of an event. A "+-" will mean that an increase in the component can increase or decrease the probability of an event.

For example, increases in unease and SMP tend to increase anxiety, and increases in PMP tend to decrease anxiety. This would be written as,

prob ( Exp ⊃ "anxiety ") = Perception ( Env | Diff, U+, SMP+, PMP-).

Third, if one or more component has a dominant influence on the probability of an event then those components will be in **bold** text.

For example, some people with performance anxiety describe their anxiety as coming from symptoms of excessive sympathetic activation even if they do not feel that uneasy about the performance itself. We would then write

prob ( Exp ⊃ "anxiety" ) = Perception ( Env ⊃ "performance" | Diff, U+, **SMP+**, PMP-).

Other people with performance anxiety describe their anxiety as dominated by unease with secondary increases in SMP. This would be written

prob ( Exp ⊃ "anxiety" ) = Perception ( Env ⊃ "performance" | Diff, **U+**, SMP+, PMP-).

The first type of performance anxiety would be more likely to respond to a beta-blocker, a medication to reduce sympathetic symptoms, SMP, than the second type in which performance anxiety is dominated by unease with secondary increases in SMP.

With the above notation the general equations for the UM-model in such situations are,

1. prob ( Exp ) = Perception ( Env | Diff, **U**, SMP, PMP, V )
2. prob ( R ) = Selection ( Exp | Diff, **U**, SMP, PMP, V )
3. prob ( Diff ~ high ) = Assessment ( Exp | U+-, SMP +-, PMP+- )
4. prob ( U ~ high ) = Appraisal ( Exp | Diff+, U+, SMP+-, PMP-)
5. prob ( SMP ~ high ) = S-activation ( Exp | Diff+, **U+**, V- )
6. prob ( PMP ~ high ) = P-activation ( Exp | U- )
7. prob (V ~ high ) = Recharging ( Exp | SMP-, PMP+ ).

The verbal meaning of the equations is,

1. The probability of a particular experience arises from a process of perception given an environment and the levels of difficulty, unease, SMP, PMP and reserves, with unease having a dominant influence.
2. The probability of a particular response arises from a process of selection given an experience and the levels of difficulty, unease, SMP, PMP, and reserves, with unease having a dominant influence.
3. The probability of difficulty being high arises from a process of assessment given an experience and the levels of unease, SMP and PMP. Increases in unease, SMP, and PMP can either increase or decrease the probability that difficulty is high.
4. The probability of unease being high arises from a process of appraisal given an experience and the levels of difficulty, unease, SMP, and PMP. Increases in difficulty and unease will increase the probability that unease is high. Increases in SMP may increase or decrease the probability that unease is high, and increases in PMP will decrease the probability that unease is high.
5. The probability of SMP being high arises from a process of S-activation given an experience and the levels of difficulty, unease and reserves. Increases in difficulty and unease will increase the probability that SMP is high with unease having the dominant influence. Increases in reserves will reduce the probability that SMP is high.
6. The probability of PMP being high arises from a process of P-activation given an experience and the level of unease. Increases in unease will reduce the probability of PMP being high.
7. The probability of reserves being high arises from a process of recharging given an experience and the levels of SMP and PMP. Increases in SMP will reduce the probability that reserves are high. Increases in PMP will increase the probability that reserves are high.

See Supplementary Figure S-2.

Note that unease appears in more equations than any other component and has a dominant effect on response. Thus, unease has particular importance and modulating unease is necessary.

In working with a patient, the initial goal is to identify the particular types of experience and response to be changed. Then, the relevant components are identified and interventions are introduced to alter those. The interactions among the components can be used to alter a relevant component indirectly.

As an example, the UM-Model will be used to analyze the development of opioid use disorder from treatment of acute pain with opioids, and determine methods to prevent the development of opioid use disorder when using opioids to treat acute pain.

The first author has extensive clinical experience with patients who were not opioid addicts and were prescribed opioid analgesics appropriate for medical conditions. Some of these patients developed opioid use disorder and some did not. None of the patients who developed opioid use disorder started taking their prescribed medication with the intent to misuse it or to become an opioid addict. He has also never worked with a physician who prescribed opioids for a patient to treat their pain with the intent of causing the patient to develop an addiction to opioids. It is puzzling how both patients and doctors can, in good faith, start on a course of treatment that leads to such a negative outcome. The UM-Model can give some insights as to how that happens.

In the initial state prior to being in pain, the patient does not desire opioids and the patient's responses to their experience have nothing to do with opioid use,

Prob ( Exp ⊃ "desire for opioids" ) = 0,

and

prob ( R ⊃ "take opioid analgesic" ) = 0.

After the patient is prescribed an opioid analgesic for pain they experience a reduction in pain after taking the opioid. Pain is aversive and since opioids reduce pain they reduce aversion and thus reduce unease. The person who uses pain medication finds that taking pain medication becomes more likely if they feel pain.

if Exp ⊃ "pain is intense",

then prob ( R ⊃ "take opioid analgesic" ) = *a* > 0.

Opioids reduce unease rapidly as well as pain. A person who is prescribed opioids for pain is likely to have unease from sources other than pain. Sometimes, after taking an opioid to relieve pain, the patient will experience a reduction in unease from other sources as well as a reduction in pain. This rapid reduction in unease from sources other than pain will occur even when the patient is legitimately using the opioid to relieve pain, see Supplementary Figure S-3. The more often the patient experiences the rapid reduction in unease from taking the opioid to relieve pain the more the patient's brain becomes conditioned to perceive unease as pain and thus use the opioid to reduce perceived pain that comes from unease, see Supplementary Figure S-4.

prob ( Exp ⊃ "pain is intense" ) increases if U ~ high,

prob( R ⊃ "take opioid analgesic" ) *b* > *a*.

This conditioning occurs without the patient being aware of it. The patient experiences an increase in pain, and then a reduction in pain after taking the opioid **as prescribed**. Note that the conditioning comes from the fact that opioids reduce unease, not from the formulation of the opioid. The UM-Model therefore predicts that "abuse deterrent" formulations of an opioid will not reduce the development of opioid use disorder in patients who are prescribed "abuse deterrent" opioid analgesics.

Even though the patient is using the opioid to reduce unease from sources other than pain, the patient does not have an opioid addiction because they are not experiencing unease from the absence of the opioid. However, the risk of developing unease due to the absence of the opioid, "absence unease", is high if use of the opioid continues. When "absence unease" first develops the person experiences unease as the opioid wears off, but perceives this as an increase in pain. Since taking the opioid reduces the "absence unease" the patient experiences a reduction in pain and continue to use the opioid to relieve pain, see Supplementary Figure S-5. The fact that "absence unease" is driving the experience of pain is not obvious to the patient or the prescriber.

prob ( Exp ⊃ "pain" ) increases if U ~ high,

U ~ high if opioid wears off due to "absence unease",

Prob ( R ⊃ "take opioid analgesic" ) ~ 1.

One very early sign of "absence unease" occurs when the patient reports increases in pain a consistent amount of time after taking the same dose of medication. Since pain and other sources of unease should vary, if pain appears consistently the same number of hours after taking the same dose of opioid then the perceived pain is at least partially from "absence unease". A later sign of "absence unease" is tolerance to the unease-reducing effects of the opioid so that increasing doses or an increasing frequency of doses are needed for relief, see Supplementary Figure S-6.

Once a patient is experiencing unease solely due to the absence of the opioid then they are meeting the second of the conditions for an addiction to opioids. The patient's opioid use disorder may be hidden but will almost certainly worsen over time and eventually lead to other unhealthy behaviors to obtain opioids that make the opioid use disorder obvious. By then, the patient may require intensive treatment for opioid use disorder and even if that is available it may not be successful.

prob (Exp ⊃ "need for opioid") ~ 1,

prob ( R ⊃ "opioid use ) ~ 1,

prob ( R ⊃ "illicit behaviors to obtain opioids" ) ~ 1.

In order to treat acute pain with opioids and reduce the risk of causing opioid use disorder it is necessary to help the patient modulate their unease without using opioids, and it is especially important minimize "absence unease".

The UM-Model can be used to help the patient modulate their unease. According to the model, what reinforces the use of the opioid analgesic is not just the reduction in pain after such use, but the large reduction in unease associated with taking the opioid.

prob( U ~ "high" ) = Appraisal( Exp ⊃ "pain" ) ~ 1,

R ⊃ "take opioid analgesic",

prob ( U ~ "low") = Appraisal( Exp ⊃ "less pain due to opioid" ) ~ 1.

If the reduction in unease can be reduced, or the reduction in unease can be associated with other responses, then the use of the opioid analgesic will be reinforced less and the chance of the patient developing opioid use disorder will be less. Two methods exist to reduce this reduction in unease. One method is to teach the patient to change their appraisal process. Another is to teach the patient to change other components of the UM-Model that will then reduce the reduction in unease.

Patients can change their appraisal process if they are educated about the inherent addictive potential of opioids, even if they are used as prescribed. This will tend to cause the patient to experience some unease when they use an opioid for pain, and that increase in unease will tend to counter the decrease in unease caused by the opioid. The unease level after taking the opioid is likely to be moderate rather than low and that will reduce the reduction in unease and thus reduce the reinforcing effect.

prob( U ~ "high" ) = Appraisal( Exp ⊃ "pain" ) ~ 1,

R ⊃ "take opioid analgesic",

prob( U ~ "moderate" ) = Appraisal( Exp ⊃ "less pain due to opioid" ) ~ 1.

Many patients report being at least somewhat uneasy about using opioids and while it may be difficult to educate all patients so that they experience sufficient unease about using opioids, it is important not to make them comfortable with the idea of taking opioid analgesics by falsely reassuring them that opioid analgesics are not addictive if taken as prescribed.

Several methods exist to change other components of the UM-Model in ways that reduce the reduction in unease associated with taking an opioid analgesic.

Recall that

prob( U ~ "high" ) = Appraisal( Exp ⊃ "pain" | Diff+, U+, SMP+-, PMP- )

Increases in PMP will tend to reduce unease. Increases in difficulty will tend to increase unease and decreases in difficulty will tend to decrease unease.

Since we need to reduce the reduction in unease associated with taking an opioid this can be done by reducing unease prior to taking an opioid and by coupling other responses to the decrease in unease.

Relaxation, mindfulness and other mental techniques can reduce unease. If the patient uses one of these techniques successfully prior to taking an opioid analgesic then unease will have decreased before the opioid analgesic is taken.

prob( U ~ "moderate" ) = Appraisal( Exp ⊃ "pain with effect of mental technique" ) ~ 1.

If the patient then takes an opioid analgesic, the reduction in unease will be from moderate to low rather than from high to low. The smaller reduction in unease will reduce the reinforcement of taking the opioid analgesic.

Obtaining a noticeable reduction in unease from mental techniques while experiencing pain requires skill in using such techniques. Not all patients will have this level of skill. However, the mental techniques can be used simultaneously with the opioid analgesic. The reduction in unease will still be large, but now the reduction is coupled with the mental technique used as well as the opioid analgesic, see Supplementary Figure S-7.

prob( U ~ "high" ) = Appraisal( Exp ⊃ "pain" ) ~ 1,

R ⊃ "take opioid analgesic and use mental technique",

prob( U ~ "low" ) = Appraisal( Exp ⊃ "less pain due to opioid and mental technique" ) ~ 1.

That will reinforce the response of the mental techniques and its effectiveness.

Responses that reduce difficulty also reduce unease. If patients are consistent with engaging in activities to either reduce demands or increase resources as the opioid takes effect, then the reduction in unease will be significant but also coupled to the activity, not just to the opioid analgesic.

prob( U ~ "high" ) = Appraisal( Exp ⊃ "pain" ) ~ 1,

R ⊃ "take opioid analgesic and act to reduce difficulty",

prob( U ~ "low" ) = Appraisal( Exp ⊃ "less pain due to opioid and activity" ) ~ 1.

This will reinforce the response of using activities to reduce difficulty.

While the use of conditional probability distributions in the UM-Model is complex, the model can be used to make clinical interventions without any reference to the underlying mathematics. In the case of prescribing opioid analgesics, the UM-Model predicts that patients who will benefit from the use of opioid analgesic can be protected from developing opioid use disorder if:

- they are prescribed the opioids for a short a time as is necessary
- they are educated to have a perceptible level of unease about using the opioid,
- they learn non-pharmacologic techniques to reduce difficulty and increase PMP, and apply those consistently when they experience pain, whether or not they take an opioid analgesic,
- prescribers realize that reports of increased pain occurring a consistent amount of time after taking an opioid dose may be an early sign the patient is developing "absence unease".

References:

Hoffman, D.D. and Prakash, C., (2014). Objects of consciousness. Frontiers in Psychology. 5, article 577, pp 1-2.

Fields, C., Hoffman, D.D., Prakash, C., and Singh, M., (2017). Conscious agent networks: Formal analysis and application to cognition. Cognitive Systems Research. 47, 186-213.
